# Proteomic and metabolomic revealed the effect of shading treatment on cigar tobacco

**DOI:** 10.3389/fpls.2024.1433575

**Published:** 2024-07-19

**Authors:** Tongjing Yan, Bin Cai, Fangyou Li, Dong Guo, Changjian Xia, Hongkun Lv, Beisen Lin, Huajun Gao, Zhaoliang Geng

**Affiliations:** Haikou cigar Research Institute, Hainan Provincial Branch of China National Tobacco Corporation, Haikou, China

**Keywords:** proteomic, metabolomic, shading treatment, cigar tobacco, biosynthesis of terpenoids, Calvin cycle

## Abstract

Shading or low light conditions are essential cultivation techniques for cigar wrapper tobacco leaves production, yet their impact on protein and metabolic regulatory networks is not well understood. In this study, we integrated proteomic and metabolomic analyses to uncover the potential molecular mechanisms affecting cigar tobacco leaves under shading treatment. Our findings include: (1) Identification of 780 significantly differentially expressed proteins (DEPs) in the cigar wrapper tobacco leaves, comprising 560 up-regulated and 220 down-regulated proteins, predominantly located in the chloroplast, cytoplasm, and nucleus, collectively accounting for 50.01%. (2) Discovery of 254 significantly differentially expressed metabolites (DEMs), including 148 up-regulated and 106 down-regulated metabolites. (3) KEGG pathway enrichment analysis revealed that the mevalonate (MVA) pathway within ‘Terpenoid backbone biosynthesis’ was inhibited, leading to a down-regulation of ‘Sesquiterpenoid and triterpenoid biosynthesis’. Conversely, the 2-C-methyl-D-erythritol 4-phosphate (MEP) pathway was enhanced, resulting in an up-regulation of ‘Monoterpenoid biosynthesis’, ‘Diterpenoid biosynthesis’, and ‘Carotenoid biosynthesis’, thereby promoting the synthesis of terpenoids such as carotenoids and chlorophylls. Simultaneously, the Calvin cycle in ‘Carbon fixation in photosynthetic organisms’ was amplified, increasing photosynthetic efficiency. These results suggest that under low light conditions, cigar tobacco optimizes photosynthetic efficiency by reconfiguring its energy metabolism and terpenoid biosynthesis. This study contributes valuable insights into protein and metabolic analyses, paving the way for future functional studies on plant responses to low light.

## 1 Introduction

Light plays a crucial role in the lifecycle of plants, acting as a key ingredient in their growth and development processes. This was emphasized by De Wit and his colleagues in 2016 (De Wit et al., 2016). Light is not only pivotal for photosynthesis, but also for the plant’s ability to produce food, demanding adaptability to the ever-changing sunlight exposure, as noted by Bukhov in 2004 (Bukhov, 2004). The ability of a plant to effectively adapt to varying light conditions is essential for its growth, development, and survival. Plants derive their energy from light; photosynthesis converts light energy into chemical energy via light-dependent reactions, a process detailed by Liang and others in 2016 (Liang and Lindblad, 2016). Insufficient light, therefore, poses a major challenge to plant growth (Lu et al., 2019), impacting their physiological traits, including morphology, chlorophyll content, photosynthetic efficiency, and the expression of various genes and proteins. This was explored in studies by Liang, Feng, Gao, and Yang between 2016 and 2020 (Liang and Lindblad, 2016; Feng et al., 2018; Gao et al., 2020; Yang et al., 2020). The costly and elusive cigar wrapper leaves, essential for the production of premium cigars, are sourced from plants cultivated in shaded conditions. These cigar wrapper leaves, renowned for their distinctive qualities, serve as the outer covering during the final stages of crafting exquisite cigars. Borges and his team in 2012 found that shading enhances the flavor quality of the leaves and prevents them from becoming too thick or nicotine-rich (Borges et al., 2012). Some scholars also proposed that low light could change the photosynthesis process of cigars by regulating chlorophyll content, chlorophyll fluorescence and gene expression (Wu et al., 2021). These shading treatments make tobacco leaves thinner and better looking, which could be used as cigar wrapper and increase the value of tobacco leaves. Despite these practices, the self-regulatory mechanisms of cigar tobacco under shaded conditions are not fully understood.

The rise of high-throughput sequencing and mass spectrometry has dramatically advanced omics technologies, encompassing genomics, transcriptomics, proteomics, and metabolomics. These methods offer a comprehensive view of gene transcription, signal transduction, protein expression, and metabolic reactions, as cited in sources (Lei et al., 2014; Hafidh et al., 2016; Li et al., 2017). Proteomics, in particular, bridges the gap between gene transcription and metabolic responses, quantifying protein levels. Integrating proteomics with transcriptomics enhances understanding of regulatory networks. Combining it with metabolomics sheds light on post-transcriptional regulation in metabolic changes, as found in studies by Liu, Haider, and Liang between 2013 and 2016 (Haider and Pal, 2013; Liang et al., 2016; Liu et al., 2019a). Metabolomics, a critical component of systems biology, identifies a wide range of endogenous metabolites and is widely used in food, tea, and tobacco research (Daglia et al., 2014; Liu et al., 2019b, 2020; Tsaballa et al., 2020; Zheng et al., 2021). Previous research, including transcriptomic and proteomic analyses, revealed complex gene and protein expression patterns in plants under stress, as Venkataramanan et al. high-lighted in 2015 (Venkataramanan et al., 2015). Studies have explored transcriptomic responses to low light in crops like rice and cherry (Sekhar et al., 2019; Tian et al., 2020), and proteomic responses in maize and rice (Liu et al., 2014; Gao et al., 2020). However, research on cigar tobacco’s proteomic and metabolomic responses to low light is limited, with a few studies addressing high light conditions and changes in cigar tobacco leaves under varying light (Schuster et al., 2020; Ma et al., 2023).

This study investigates the adaptive mechanisms and regulatory responses of cigar tobacco to low light. Using proteomic and metabolomic analyses, we aim to uncover the changes in proteins and metabolites in cigar tobacco leaves under these conditions. Our approach seeks to provide new insights into the plant’s response and physiological adaptations to light stress, enhancing our understanding of plant resilience in suboptimal light environments.

## 2 Materials and methods

### 2.1 Plant materials and growth conditions

For our study, we selected the Haiyan 103 variety of *Nicotiana tabacum L.*, grown in Danzhou City, Hainan Province. We used the same variety for both shaded (ST) and non-shaded treatments (NST) to ensure consistency. In line with local guidelines for quality cigar tobacco cultivation, each treatment was given 180 Kg of pure nitrogen, 270 Kg of P_2_O_5_, and 360 Kg of K_2_O per hectare. Planting commenced on January 8, 2023, and 66 square meters were planted for ST and NST groups, respectively. Shading started on February 8, 2023. The shading net material is white polyethylene, and the transparency of the shading net is 75%. Sampling from both ST and NST was conducted on March 15, 2023. At the time of sampling, eight plants were randomly selected from each group as a biological duplicate sample. The 7th leaf on cigar tobacco plants (counting from the base to the tip, intact leaves with an area of around 48 × 26 cm) was selected for sampling, and the main vein of leaf was removed. The leaves were pooled, wrapped in foil, and flash-frozen in liquid nitrogen. Three independent biological replicates were used for proteomic analysis, while six independent biological replicates were used for metabolomic analysis. For each independent biological replicate, we conducted sufficient freezing and grinding with liquid nitrogen during sampling to ensure the uniformity of the sample.

### 2.2 Proteomic analysis

#### 2.2.1 Protein isolation and digestion process

Following Wu et al.’s methodology (Wu et al., 2020), protein isolation began with grinding 0.5 g of tobacco leaf tissue into powder in liquid nitrogen. The powder was then processed with a lysis buffer (50 mM Tris-HCl, pH 8, 8 M urea, 0.2% Sodium dodecyl sulfate). To break disulfide bonds, we added 10 mM dithiothreitol to the mixture and incubated it at 56°C for one hour. Afterward, we added iodoacetic acid and let the mixture sit for another hour in a dark environment at room temperature. The samples were then mixed with four times their volume of cold acetone, vortexed, and left at −20°C overnight. Following centrifugation (12000 × g, 4°C for 5 min), we washed the sample twice with cold acetone (4 × volume), dissolved them in a solution of 0.1 M Triethylammonium bicarbonate (TEAB, pH 8.5) and 8 M urea, and measured the protein concentration using the Bradford method.

Protein digestion was carried out as described by Zhang et al (Zhang et al., 2016). We diluted each protein sample to 100 μL using DB lysis buffer (8 M urea, 100 mM TEAB, pH 8.5), then added trypsin and 100 mM TEAB, incubating it at 37°C for four hours. Further trypsin and CaCl_2_ were added for overnight digestion. Formic acid was mixed with digested sample, adjusted pH to 3, and centrifuged the sample at 12000 × g for 5 min at room temperature. The supernatant was slowly loaded onto the C_18_ desalting column, washed 3 times with washing buffer (0.1% formic acid, 3% acetonitrile), and then eluted with the elution buffer (0.1% formic acid, 70% acetonitrile). The eluates were then lyophilized for analysis.

#### 2.2.2 Proteomic analysis via UHPLC-MS/MS

For proteomic analysis, we used an Easy-nLC™ 1200 UHPLC and a Q Exploris™ HF-X mass spectrometer. We loaded 4 μg of each sample, mixed with iRT reagent, onto a C_18_ Nano-Trap column. The gradient profile ranged from 5% to 95% acetonitrile in formic acid over 92 min, with a flow rate of 600 nL/min. The peptides underwent analysis by a Q Exactive™ HF-X mass spectrometer, with specific settings for full scan range, resolution, AGC target, ion injection time, and fragmentation settings. The top 40 precursors were selected for MS/MS analysis, and DIA mode was utilized for broader peptide coverage.

#### 2.2.3 Identification and quantification of proteins

We independently searched the spectra from each fraction against the Nicotiana tabacum protein database using Proteome Discoverer 2.2. The search parameters were finely tuned for precision, and the proteins identified needed to meet stringent criteria including FDR and amino acid coverage.

#### 2.2.4 Data analysis and statistical approaches

For functional annotation, we utilized Gene Ontology and InterPro analyses via InterProScan, analyzing against a comprehensive protein database. We also employed COG and KEGG databases for further analysis of protein families and pathways. Differential protein expression was assessed using various analytical tools, including Volcano plot and heat map analysis. Protein-protein interactions were predicted using the STRING-db server.

### 2.3 Metabolomic analysis

#### 2.3.1 Process of metabolite extraction and untargeted metabolomic analysis

We began by grinding 0.1 g of leaf tissue in liquid nitrogen to create a fine powder. This powder was then reconstituted in 80% methanol that had been chilled beforehand. After vigorous shaking of the mixture, it was left on ice for 5 min before being subjected to centrifugation at 15,000 × g at a temperature of 4°C, lasting for 20 min. We then diluted a part of the clear supernatant to achieve a concentration of 53% methanol, utilizing water of UHPLC-MS/MS grade. The diluted mixture was placed into new Eppendorf tubes and centrifuged once more under identical conditions. The final supernatant obtained from this process was then prepared for analysis via UHPLC-MS/MS.

The analysis of the samples was conducted using a Vanquish UHPLC system from ThermoFisher (Germany), in conjunction with an Orbitrap Q ExactiveTM HF mass spectrometer. Samples were injected onto a Hypersil Gold column (100 × 2.1 mm, 1.9 μm) using a 12 min linear gradient at a flow rate of 0.2 mL/min. The eluents for the positive polarity mode were eluent A (0.1% Formic acid in Water) and eluent B (Methanol). The eluents for the negative polarity mode were eluent A (5 mM Ammonium acetate, pH 9.0) and eluent B (Methanol). The solvent gradient was set as follows: 2% B, 1.5 min; 2–85% B, 3 min; 85–100% B, 10 min; 100–2% B, 10.1 min; 2% B, 12 min. The mass spectrometer settings, including spray voltage, capillary temperature, gas flow rates, and S-lens RF level, were finely adjusted. The MS scan covered a range from 90 to 900 m/z, utilizing fragmentation data acquisition methods in both polarity modes.

#### 2.3.2 Processing and analysis of metabolomic data

For the processing of metabolomics data, we used Compound Discoverer 3.3, following the approach described by Ribbenstedt et al (Ribbenstedt et al., 2018). The metabolite identification process involved querying the acquired data against an array of databases such as mzCloud, mzVault, KEGG, and several others, maintaining a precise mass tolerance. For the purpose of annotating these metabolites, we utilized well-known databases like KEGG, HMDB, and LIPID Maps, each offering a wealth of information for accurate metabolite identification and classification.

### 2.4 Synthesis of proteomic and metabolomic data

The integration of the identified DEPs and DEMs into the KEGG pathway maps facilitated the visualization of alterations in crucial metabolic pathways. We focused on pathways including ‘Terpenoid backbone biosynthesis’, and others related to terpenoid synthesis, as well as the ‘Carbon fixation in photosynthetic organisms’ pathway to depict the Calvin cycle. This integration helped in constructing a detailed representation of the terpenoid biosynthesis process.

## 3 Results

### 3.1 Comparative analysis of protein profiles in ST and NST groups

#### 3.1.1 Multivariate statistical evaluation of protein profiles

**Figure 1:**
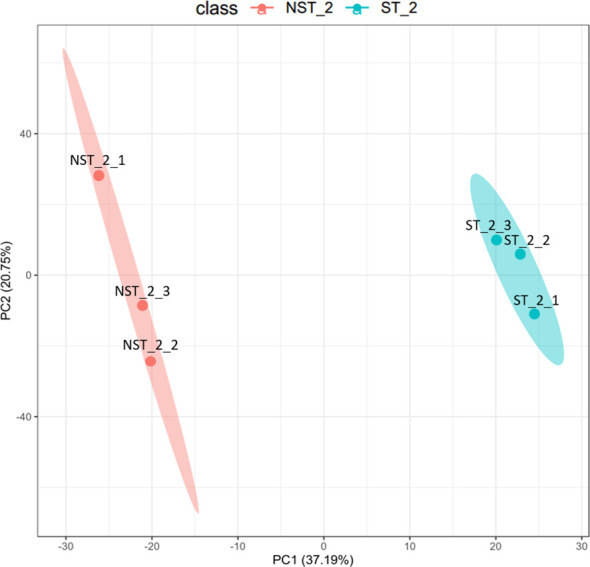
PCA diagram of cigar tobacco leaves in ST and NST groups. The abscissa PC1 and the ordinate PC2 in the figure represent the scores of the first and second principal components, respectively. The ellipse is the 95% confidence interval. The sample number of NST_2 is NST_2_1~3, and the sample number of ST_2 is ST_2_1~3.

The unsupervised, multi-factor principal component analysis (PCA) method was used to analyze the protein profile data of cigar tobacco leaves treated with shading and non-shading. The results are shown in **Figure 1**. The two groups of cigar tobacco leaves were significantly separated along the first principal component, while the dispersion trend between samples within each treatment group was not obvious and all samples were within the 95% confidence interval, indicating that there were significant differences between the protein profile of cigar tobacco leaves in ST and NST groups.

#### 3.1.2 Examination of DEPs in cigar tobacco leaves

**Figure 2:**
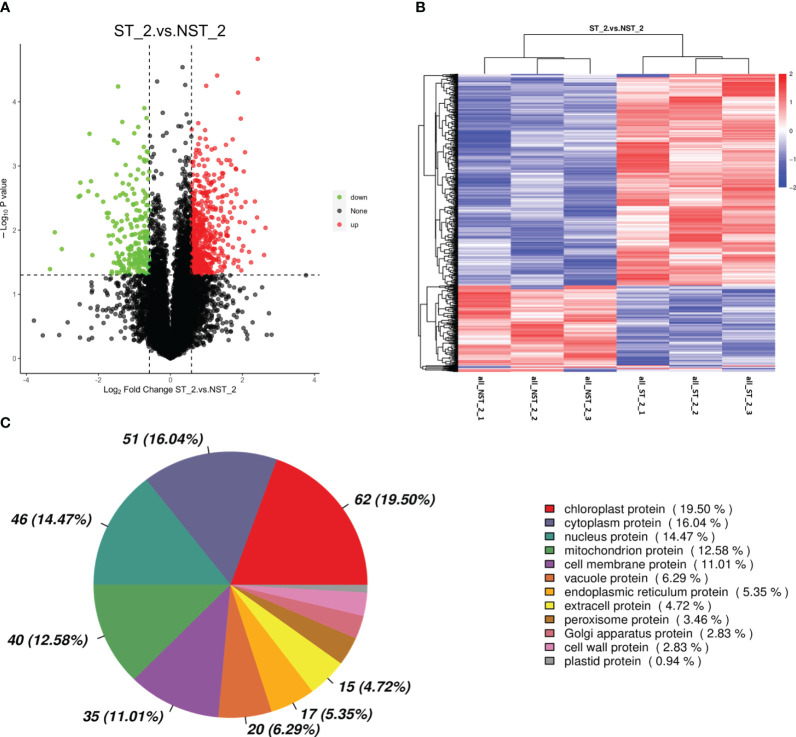
Volcano map, cluster heat map and subcellular localization of DEPs of cigar tobacco leaves in ST and NST groups. **(A)** Abscissa represents the multiple change of expression of proteins in different groups (log_2_ Fold Change), ordinate indicates the significant level of difference (-log_10_ P-value), each point in the volcanic map represents a protein, significantly up-regulated proteins are represented by red dots, significantly down-regulated proteins are represented by green dots. **(B)** ST_2_1–3 and NST_2_1–3 represent the six repeated samples for the analysis of proteomic of ST and NST groups, respectively. **(C)** Location of DEPs in cells.

The proteomic of cigar tobacco leaves in ST and NST groups allowed us to quantify a total of 12553 proteins. In this study, all proteins were screened and analyzed using a threshold of VIP > 1, FC > 1.5 or FC < 0.67, *P* < 0.05. The results showed that compared to NST group, cigar tobacco leaves in ST group included 780 DEPs, of which 560 DEPs were up-regulated and 220 DEPs were down-regulated (**Figure 2A**; **Supplementary Table S1**). The heatmap analysis was conducted on 780 DEPs of cigar tobacco leaves in ST and NST groups (**Figure 2B**). The red color in heatmap represents DEPs with higher than average content, while the blue color represents DEPs with lower than average content. Comparing the intensity of all colors, it was found that most DEPs in the same treatment group showed similar patterns. According to Pearson correlation coefficients, all samples can be divided into two categories: ST and NST. This is similar to the results of PCA, indicating that there are significant differences in proteins of cigar tobacco leaves between ST and NST groups. As shown in **Figure 2C** and **Supplementary Table S2**, subcellular localization analysis found that most of DEPs of cigar tobacco leaves in ST and NST groups were located in chloroplast (19.50%), cytoplasm (16.04%), and nucleus (14.47%), accounting for 50.01%.

### 3.2 Comparative metabolite analysis in ST and NST groups

#### 3.2.1 Multivariate statistical evaluation of metabolite profiles

**Figure 3:**
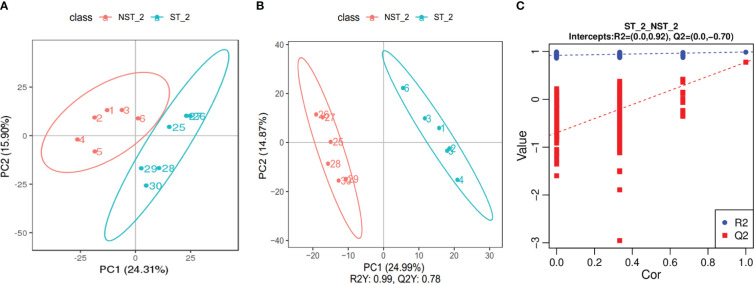
PCA diagram, PLS-DA score scatter diagram and sequencing verification diagram of cigar tobacco leaves in ST and NST groups. **(A)** The abscissa PC1 and the ordinate PC2 in the figure represent the scores of the first and second principal components, respectively. The ellipse is the 95% confidence interval. The sample number of NST_2 is 1~6, and the sample number of ST_2 is 25~30. **(B)** The abscissa is the score of the sample on the first principal component, the ordinate is the score of the sample on the second principal component. The sample number of NST_2 is 1~6, and the sample number of ST_2 is 25~30. **(C)** The abscissa represents the correlation between the Y of the random grouping and the original group Y, and the ordinate represents the scores of R^2^ and Q^2^.

The PCA results are shown in **Figure 3A**. The samples from ST and NST groups were significantly separated along the first and second principal components, while the dispersion trend between samples within each treatment group was not obvious and all samples were within the 95% confidence interval, indicating significant differences between the metabolite profiles of cigar tobacco leaves in the two treatment groups. The partial least squares discriminant analysis (PLS-DA) is shown in **Figure 3B**. The two groups of cigar samples are clearly separated in the PLS-DA score diagram. In the PLS-DA model, the first two components R2 Y and Q2 Y dominate, with values of 99% and 78%, respectively, indicating that the model has good stability and that the selection of the first two components is sufficient to test the dataset. In addition, permutation testing was used to evaluate whether the PLS-DA model was ‘overfitting’. As shown in **Figure 3C**, R^2^ was 0.92 and Q^2^ was -0.70. R^2^>Q^2^ and the intercept of the Q^2^ regression line with the Y axis was less than 0, indicating that the PLS-DA model was not ‘overfitting’ and could better describe the classification of samples, which indicating that ST had a significant impact on the metabolite profile of cigar tobacco leaves.

#### 3.2.2 Exploration of DEMs between ST and NST groups

**Figure 4:**
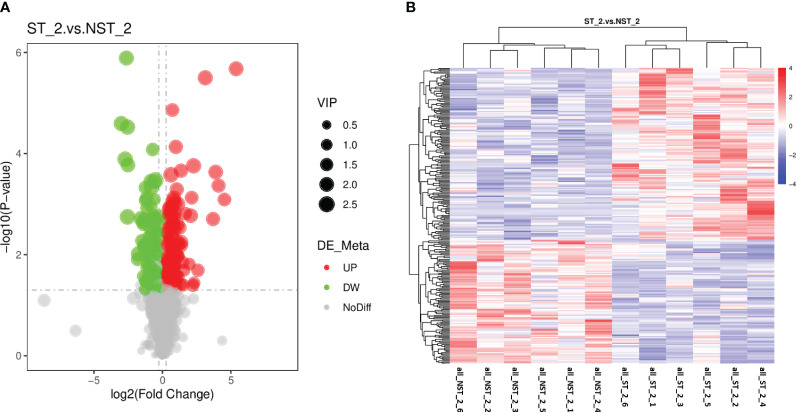
Volcano map and cluster heat map of DEMs of cigar tobacco leaves in ST and NST groups. **(A)** Abscissa represents the multiple change of expression of metabolites in different groups (log_2_ Fold Change), ordinate indicates the significant level of difference (-log_10_ P-value), each point in the volcanic map represents a metabolite, significantly up-regulated metabolites are represented by red dots, significantly down-regulated metabolites are represented by green dots, and the size of dots represents VIP values. **(B)** ST_2_1–6 and NST_2_1–6 represent the six repeated samples for the analysis of metabolomic of ST and NST groups, respectively.

The metabolic of cigar tobacco leaves in ST and NST groups allowed us to quantify a total of 1163 metabolites. In this study, all metabolites were screened and analyzed using a threshold of VIP > 1, FC > 1.2 or FC < 0.83, *P* < 0.05. The results showed that compared to NST group, cigar tobacco leaves in ST group included 254 DEMs, of which 148 DEMs were up-regulated and 106 DEMs were down-regulated (**Figure 4A**; **Supplementary Table S3**). The heatmap analysis was conducted on 254 DEMs of cigar tobacco leaves in ST and NST groups (**Figure 4B**). The red color in heatmap represents DEMs with higher than average content, while the blue color represents DEMs with lower than average content. Comparing the intensity of all colors, it was found that most DEMs in the same treatment group showed similar patterns. According to Pearson correlation coefficients, all samples can be divided into two categories: ST and NST. This is similar to the results of PCA and PLS-DA, indicating that there are significant differences in metabolites of cigar tobacco leaves between ST and NST groups.

### 3.3 Merging proteomic and metabolomic data studies

**Figure 5:**
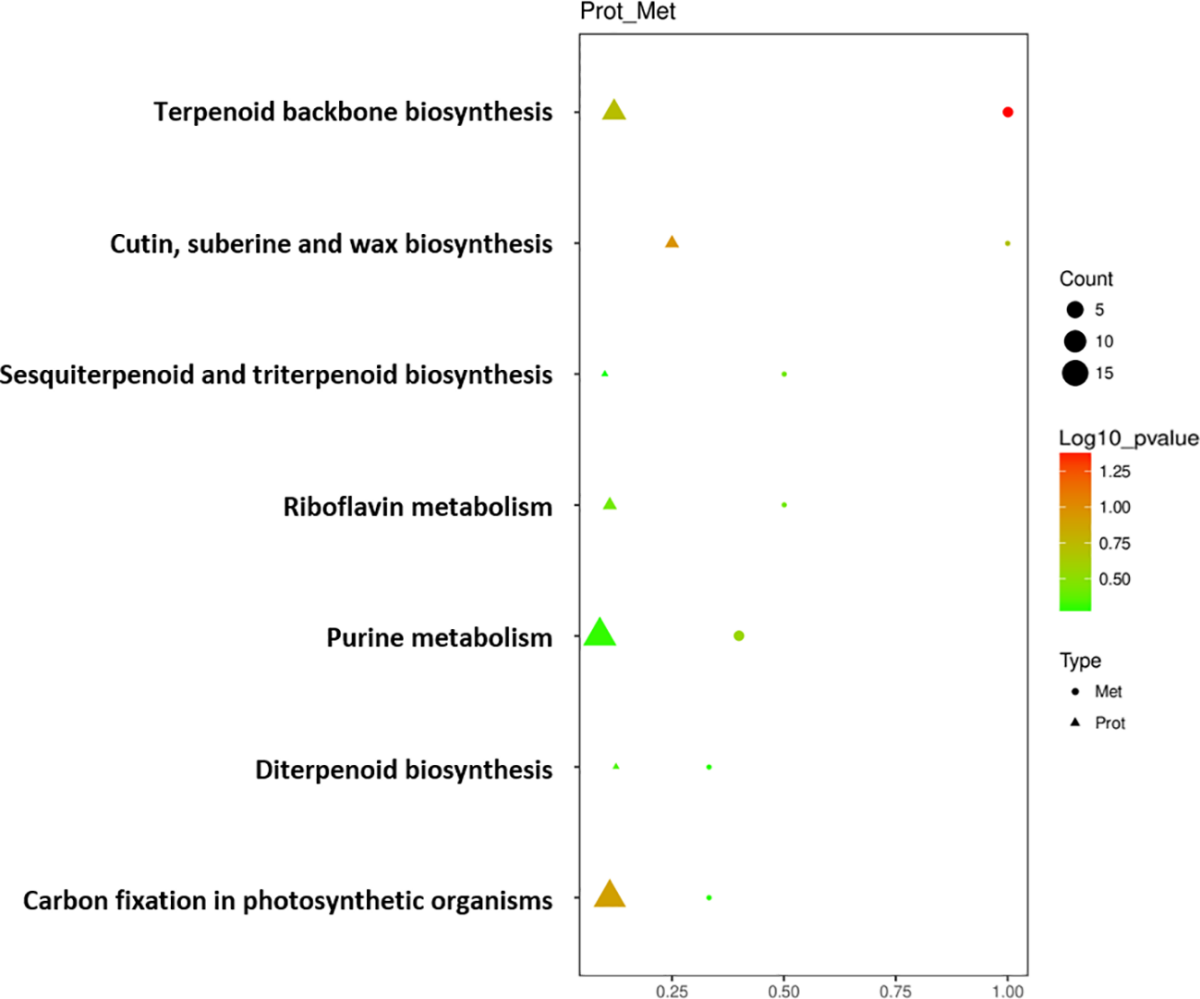
Bubble diagram of enrichment pathway of DEPs and DEMs of cigar tobacco leaves in ST and NST groups. Protein was represented by triangles, metabolite was represented by circles. Horizontal axis indicates the enrichment degree of DEPs and DEMs. Point color represents the P-value, and point size indicates the number of DEPs and DEMs in the corresponding pathway.

Seven key pathways were obtained through KEGG enrichment pathway association analysis of DEPs and DEMs screened out above (**Figure 5**). DEPs and DEMs were mainly enriched in the pathways of ‘Terpenoid backbone biosynthesis’, ‘Cutin, suberine and wax biosynthesis’, ‘Sesquiterpenoid and triterpenoid biosynthesis’, ‘Riboflavin metabolism’, ‘Purine metabolism’, ‘Diterpenoid biosynthesis’, and ‘Carbon fixation in photosynthetic organisms’. Among them, ‘Terpenoid backbone biosynthesis’, ‘Sesquiterpenoid and triterpenoid biosynthesis’ and ‘Diterpenoid biosynthesis’ pathways refer to the process of synthesizing terpenoids such as chlorophyll and carotenoids in cigar tobacco leaves through biochemical pathways; ‘Carbon fixation in photosynthetic organisms’ pathway refers to the process of converting atmospheric carbon dioxide into carbon in its own organic matter through photosynthesis in cigar tobacco leaves; ‘Cutin, suberine and wax biosynthesis’ pathway refers to the process of combining long-chain fatty acids and alcohol compounds through biochemical pathways to form substances such as cutin, suberin and wax that constitute the cell structure of cigar tobacco leaves; ‘Riboflavin metabolism’ and ‘Purine metabolism’ pathways refer to the process of synthesizing and decomposing riboflavin and purine in cigar tobacco leaves through biochemical pathways.

**Figure 6:**
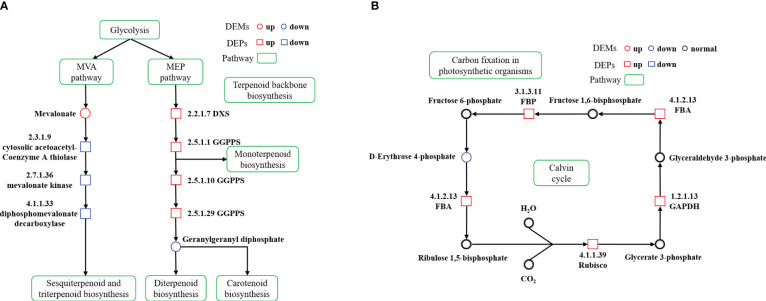
Terpenoid biosynthesis pathway diagram and differences in protein and metabolite content **(A)** Calvin cycle diagram and differences in protein and metabolite content **(B)** in cigar tobacco leaves. The proposed metabolic pathways were based on KEGG PATHWAY Database. The red represents high relative content and the blue represents low relative content. The green represents metabolic pathway.

Through association analysis of the enriched metabolic pathways (**Figure 6**), a terpenoid biosynthesis process diagram was obtained by combining the metabolic pathways of ‘Terpenoid backbone biosynthesis’, ‘Sesquiterpenoid and triterpenoid biosynthesis’, ‘Monoterpenoid biosynthesis’, ‘Diterpenoid biosynthesis’ and ‘Carotenoid biosynthesis’. Calvin cycle diagram was also drawn based on the metabolic pathway of ‘Carbon fixation in photosynthetic organisms’.

## 4 Discussion

Light’s transformation into biochemical energy is vital in plant growth, playing an indispensable role in their development, as highlighted by Evans (Evans, 2013). In tobacco plants, shading impedes the availability of light, diminishing the rate of photosynthesis in leaves, leading to less material accumulation and thinner leaves, a phenomenon documented by Wu et al. (2021). Plants, however, have developed adaptive mechanisms to combat low-light stress, as detailed in the works of Gao et al (Gao et al., 2020). and Tian et al (Tian et al., 2020). Adopting a multi-faceted data integration approach, encompassing genetic, epigenetic, transcriptomic, and proteomic perspectives, can shed light on plant responses to various non-living stresses, a concept explored by Haak et al (Haak et al., 2017). It’s critical to recognize, as Liu et al. point out, that protein levels may not always be indicative of metabolite abundance (Liu et al., 2016). Sole reliance on a single methodology, like proteomics or metabolomics, risks partial or incorrect interpretations of the underlying processes. Thus, merging various omics methods is crucial for an accurate interpretation, as emphasized by Zapalska-Sozoniuk et al (Zapalska-Sozoniuk et al., 2019). In our research, shading treatment (ST) revealed significant variances in differentially expressed proteins (DEPs) and metabolites (DEMs). However, a direct correlation between the proteomic and metabolomic data was absent. This inconsistency could stem from factors like delayed metabolite reactions. Ding et al. noted that proteins react more swiftly to environmental shifts than metabolites, despite using identical plant samples and treatments (Ding et al., 2020).

### 4.1 The impact of shading treatment on terpenoid synthesis in cigar tobacco leaves

Terpenoids, a vast class of over 55,000 natural secondary metabolites, are vital in plant primary and secondary metabolism (Christianson, 2017; Pu et al., 2021). These compounds are synthesized through two distinct pathways: MVA and MEP pathways. Monoterpenes, diterpenes, and carotenoids typically originate from the MEP pathway, while sesquiterpenes and triterpenes are produced via the MVA pathway (Moser and Pichler, 2019; Chen et al., 2020). In the ST group, we noticed an upregulation of enzymes like geranylgeranyl diphosphate synthase (GGPPS, 2.5.1.1, 2.5.1.10, 2.5.1.29) and 1-deoxy-D-xylulose-5-phosphate synthase (DXS, 2.2.1.7) in the MEP pathway. On the contrary, cytosolic acetoacetyl-coenzyme A thiolase (2.3.1.9), mevalonate kinase (2.7.1.36) and diphosphomevalonate decarboxylase (4.1.1.33) in the MVA pathway were significantly downregulated, with an increase in mevalonic acid. These findings suggest a suppression of the MVA pathway and an enhancement of the MEP pathway, facilitating an increased conversion of glycolysis metabolites through the MEP pathway. The GGPPS in cigar tobacco promoted the further conversion of GGPP to terpenoids such as chlorophyll and carotenoids, thus enabling cigar tobacco leaves to adapt to the stress caused by shading (**Figure 6A**).

The MEP pathway starts with the transketolase decarboxylation of pyruvate and glyceraldehyde 3-phosphate (GA-3P), catalyzed by DXS, leading to 1-Deoxy-D-xylulose-5-phosphate (DXP). Subsequent reactions yield precursors for various isoprenoids, including centellosides. DXS, being the initial and flux-controlling enzyme, plays a critical role in isoprenoid biosynthesis (Estevez et al., 2001; Xiang et al., 2007; Zhang et al., 2018). However, DXS expression doesn’t always correlate directly with terpenoid levels. For instance, Umaima showed that over-expressing cyanobacterial DXS in chloroplasts disrupted the chlorophyll-carotenoid balance in Chlamydomonas reinhardtii (Hoqani et al., 2022). Erpenoids in plants start from isopentenyl diphosphate (IPP) and dimethylallyl diphosphate (DMAPP). Compounds like geranyl diphosphate (GPP), farnesyl diphosphate (FPP), and geranyl-geranyl diphosphate (GGPP) evolve from these precursors. GGPP is particularly crucial for various terpenoids like diterpenes, gibberellins, a-tocotrienol, chlorophyll, and carotenoids (Zhou et al., 2017; Dong et al., 2022). GGPPS is instrumental in converting FPP to GGPP, earmarking it for specific terpenoid biosynthesis (Ruiz-Sola et al., 2016; Akhtar et al., 2017). In Arabidopsis, the enzyme AtGGPPS11, a critical isozyme, collaborates with enzymes involved in the biosynthesis of vital compounds such as carotenoids, chlorophyll, and plastoquinone. This includes interactions with enzymes like phytoene synthase (PSY), geranylgeranyl reductase (GGR), and solanesyl diphosphate synthase 2 (SPS2) (Walter and Strack, 2011; Ruiz-Sola et al., 2016). Similarly, in rice, the enzyme OsGGPPS1 plays a pivotal role in the biosynthesis of chlorophyll, leading to enhanced plant size and increased carotenoid levels (Zhou et al., 2017). In pepper, the enzyme CaGGPPS1 is noted for its specific interaction with PSY, directing the flow of GGPP towards carotenoid production (Wang et al., 2018). Tobacco plants have shown a unique adaptation, where the enzyme NtGGPPS1, functioning as a homodimer, works alongside NtPSY1 in chloroplasts to boost carotenoid biosynthesis, a pathway that differs from AtGGPPS11’s broader role in terpenoid biosynthesis (Ruiz-Sola et al., 2016; Zhou et al., 2017). Advancements in this field include the design of a modified version of NtGGPPS1, as developed by Dong et al (Dong et al., 2022). This engineered enzyme has demonstrated a significant increase in carotenoid levels, photosynthetic efficiency, and biomass in tobacco plants. The improvement in biomass can be attributed to enhanced metabolic pathways linked to carotenoid biosynthesis, which includes the production of photoprotective molecules like β-carotene, zeaxanthin, and violaxanthin. These molecules contribute to improved photoprotection, leading to enhanced photosynthetic efficiency and biomass (Moreno et al., 2020; Kossler et al., 2021).

### 4.2 The impact of shading treatment on photosynthesis in cigar tobacco leaves

The Calvin cycle, a crucial carbon metabolism process in higher plants, operates in the chloroplast matrix and serves as the primary mechanism for CO_2_ assimilation during the dark reaction stage of photosynthesis. This cycle produces carbon compounds essential for plant growth and development (Raines, 2003; Smith and Stitt, 2007; Anoman et al., 2016). In our study, we observed a significant upregulation of proteins and metabolites related to the ‘Carbon fixation in photosynthetic organisms’ pathway under low light conditions. Key enzymes in the Calvin cycle, such as ribulose bisphosphate carboxylase (Rubisco, 4.1.1.39), glycer-aldehyde-3-phosphate dehydrogenase (GAPDH, 1.2.1.13), and fructose-bisphosphate aldolase (FBA, 4.1.2.13) and fructose-bisphosphatase (FBP, 3.1.3.11) were found to be increased, while levels of D-erythrose 4-phosphate were decreased (**Figure 6B**). Rubisco, a rate-limiting enzyme in the Calvin cycle, plays a critical role in converting atmospheric CO_2_ into energy storage molecules (Cheng et al., 1998; Portis and Parry, 2007). Despite its low catalytic efficiency, enhancing Rubisco’s activity is seen as a key strategy to improve plant photosynthetic carbon assimilation efficiency (Wang et al., 2014; Wei et al., 2022). GAPDH, an enzyme with multiple isoforms, operates in both glycolysis and the Calvin cycle, playing a dual role in energy production and CO_2_ fixation into carbohydrates (El Kadmiri et al., 2014; Anoman et al., 2016; Yu et al., 2020). FBA, a crucial enzyme in the RuBP regeneration stage of the Calvin cycle, significantly influences carbon fixation and flow (Zeng et al., 2014; Liang and Lindblad, 2016). Overexpression of FBA genes has been linked to improved plant growth and metabolism (Uematsu et al., 2012). FBP, a key enzyme in gluconeogenesis and sucrose synthesis, catalyzes the conversion of fructose 1,6-bisphosphate into fructose 6-phosphate and inorganic phosphate (Miyagawa et al., 2001). Enhancements in photosynthetic efficiency have been achieved by introducing modified versions of this enzyme into plant chloroplasts (De Porcellinis et al., 2018; Siddiqui et al., 2022). Our study found that key enzymes of the Calvin cycle were up-regulated under low light conditions, while the down-regulation of D-erythrose 4-phosphate suggests an efficient operation of this cycle. These findings indicate that cigar tobacco plants can adapt to low light conditions by reorganizing their energy metabolism, enabling them to endure stress more effectively (Thalmann and Santelia, 2017; Hwang et al., 2020).

## 5 Conclusion

In this study, we demonstrated that the changes in the intrinsic proteins and metabolites of cigar tobacco leaves are significantly related to the decrease in light intensity, manifested as the promotion of the biosynthesis of terpenoids such as carotenoids and chlorophyll, as well as the enhancement of the Calvin cycle in the “carbon fixation of photosynthetic organisms” pathway. This is the result of plants adjusting their energy requirements to compensate for the stress caused by shading, allowing plants to more effectively harvest light. This study offers novel insights into both proteomic and metabolomic processes as response mechanisms under different light intensities in cigar tobacco plants. In subsequent studies, we could combine transcriptomic and enzymatic activity assays to further clarify the impact of changes in key enzymes and metabolites on cigar tobacco leaves undergoing shading treatment.

## Data availability statement

The original contributions presented in the study are included in the article/**Supplementary Material**. Further inquiries can be directed to the corresponding authors.

## Author contributions

TY: Conceptualization, Data curation, Formal analysis, Methodology, Software, Writing – original draft, Writing – review & editing. BC: Data curation, Methodology, Writing – review & editing. FL: Conceptualization, Writing – review & editing. DG: Data curation, Methodology, Software, Writing – original draft. CX: Methodology, Software, Writing – original draft. HL: Methodology, Writing – original draft. BL: Conceptualization, Writing – original draft. HG: Formal analysis, Writing – review & editing. ZG: Formal analysis, Writing – review & editing.
