# Supplementary material for: Proteomic and metabolomic revealed the effect of shading treatment on cigar tobacco
Source: Front Plant Sci. 2024 Jul 19;15:1433575. doi: 10.3389/fpls.2024.1433575 (PMC11294240; doi:10.3389/fpls.2024.1433575)
Supplement: Supplementary file 1 [file Table_1.docx]

Table S1 DEPs identification of cigar tobacco leaves in ST and NST groups

| Protein | FC | Pvalue | log_2_FC |
| --- | --- | --- | --- |
| A0A1S4CM83 | 0.09853 | 0.04047 | -3.34321 |
| A0A1S4CHD1 | 0.10795 | 0.01080 | -3.21146 |
| A0A1S4D9K9 | 0.12359 | 0.01973 | -3.01632 |
| A0A1S4DNE4 | 0.17104 | 0.00301 | -2.54754 |
| A0A1S4CW01 | 0.17599 | 0.00291 | -2.50638 |
| A0A140G1T1 | 0.17674 | 0.00181 | -2.50023 |
| A0A1S3X8P7 | 0.20578 | 0.00247 | -2.28080 |
| A0A1S3X353 | 0.21076 | 0.00031 | -2.24629 |
| A0A1S3XBX6 | 0.21671 | 0.00173 | -2.20613 |
| A0A1S3XBU5 | 0.22264 | 0.02455 | -2.16720 |
| A0A1S3YFH7 | 0.24383 | 0.00284 | -2.03605 |
| A0A1S4A130 | 0.27406 | 0.00360 | -1.86741 |
| A0A1S3XMC3 | 0.29388 | 0.00541 | -1.76666 |
| A0A1S3XHN6 | 0.29858 | 0.00640 | -1.74379 |
| A0A1S3XTK5 | 0.30479 | 0.01219 | -1.71411 |
| A0A1S4DFK1 | 0.30541 | 0.00542 | -1.71117 |
| A0A1S3XW43 | 0.31696 | 0.01616 | -1.65761 |
| A0A1S3YZ60 | 0.32226 | 0.04773 | -1.63367 |
| A0A1S4C6X4 | 0.32357 | 0.04416 | -1.62781 |
| Q9LW94 | 0.32983 | 0.01203 | -1.60020 |
| A0A1S3XKN9 | 0.33124 | 0.00727 | -1.59405 |
| A0A1S4C9M1 | 0.33628 | 0.03199 | -1.57223 |
| A0A1S4DBY6 | 0.33766 | 0.00040 | -1.56636 |
| A0A1S4B5W5 | 0.34424 | 0.00873 | -1.53849 |
| A0A1S4C5Q2 | 0.34834 | 0.00310 | -1.52142 |
| A0A1S3Y7D9 | 0.35424 | 0.00136 | -1.49717 |
| A0A077DBL0 | 0.35501 | 0.00781 | -1.49405 |
| A0A1S4ALF1 | 0.35522 | 0.03291 | -1.49318 |
| A0A1S3X0K0 | 0.35541 | 0.02273 | -1.49242 |
| A0A1S3Z829 | 0.35670 | 0.04521 | -1.48721 |
| A0A1S3X2Z5 | 0.36200 | 0.00043 | -1.46592 |
| A0A097BU08 | 0.36241 | 0.01412 | -1.46427 |
| Q9FXS2 | 0.36514 | 0.00001 | -1.45344 |
| A0A1S4DM17 | 0.36761 | 0.02569 | -1.44373 |
| A0A0K1DA86 | 0.37003 | 0.03023 | -1.43428 |
| A0A1S3ZLL0 | 0.37009 | 0.02637 | -1.43404 |
| A0A1S4CWT7 | 0.37604 | 0.04619 | -1.41103 |
| A0A1S3Z627 | 0.37693 | 0.01005 | -1.40760 |
| A0A1S3XAM4 | 0.37847 | 0.03109 | -1.40171 |
| O82151 | 0.37852 | 0.00286 | -1.40153 |
| A0A1S4DD23 | 0.37974 | 0.01517 | -1.39690 |
| A0A1S4CDQ0 | 0.38146 | 0.03502 | -1.39039 |
| A0A1S4CXY3 | 0.38574 | 0.03404 | -1.37430 |
| A0A1S3XRR4 | 0.38989 | 0.04212 | -1.35883 |
| A0A1S3Y6I9 | 0.39045 | 0.00722 | -1.35678 |
| A0A1S3XHX8 | 0.39216 | 0.00953 | -1.35049 |
| A0A1S3ZRE0 | 0.39582 | 0.02366 | -1.33705 |
| A0A1S3Y1J2 | 0.39595 | 0.03961 | -1.33660 |
| A0A1S4DLA2 | 0.39621 | 0.00020 | -1.33566 |
| A0A1S4A5R5 | 0.39638 | 0.02330 | -1.33501 |
| A0A1S4A741 | 0.39926 | 0.00184 | -1.32458 |
| A0A1S3Y5Y3 | 0.40072 | 0.00266 | -1.31932 |
| A0A0R4WFE9 | 0.40123 | 0.03602 | -1.31747 |
| A0A1S4AXQ5 | 0.40552 | 0.04203 | -1.30213 |
| A0A1S3YFY8 | 0.40930 | 0.03914 | -1.28875 |
| A0A2H4X1W3 | 0.41283 | 0.02866 | -1.27636 |
| A0A1S4ADC3 | 0.41410 | 0.01266 | -1.27192 |
| A0A1S3Y7C7 | 0.41862 | 0.04544 | -1.25629 |
| A0A1S4D3X7 | 0.42232 | 0.00096 | -1.24356 |
| A0A1S4C7J3 | 0.42381 | 0.00282 | -1.23849 |
| A0A1S4BDU3 | 0.43298 | 0.00580 | -1.20761 |
| P13046 | 0.43620 | 0.00677 | -1.19691 |
| A0A076L1Y9 | 0.43854 | 0.00338 | -1.18921 |
| A0A1S3YIW7 | 0.44084 | 0.04488 | -1.18166 |
| A0A1S3YST1 | 0.44085 | 0.01924 | -1.18163 |
| A0A1S3ZAM8 | 0.44538 | 0.04353 | -1.16686 |
| A0A1S4C0D9 | 0.44860 | 0.00193 | -1.15650 |
| A0A1S4B8D5 | 0.44870 | 0.02971 | -1.15616 |
| A0A1S4AL11 | 0.45117 | 0.03364 | -1.14825 |
| A0A1S3YW75 | 0.45130 | 0.03968 | -1.14783 |
| A0A1S3YG04 | 0.45218 | 0.02021 | -1.14500 |
| A0A1S3XTH4 | 0.45492 | 0.02393 | -1.13629 |
| A0A1S3Y4F3 | 0.45616 | 0.02710 | -1.13238 |
| A0A1S4CDG7 | 0.46182 | 0.02437 | -1.11460 |
| A0A1S4A6V6 | 0.46424 | 0.00804 | -1.10703 |
| Q8SF02 | 0.46462 | 0.00139 | -1.10587 |
| A0A1S3YPB7 | 0.46468 | 0.04876 | -1.10569 |
| A0A1S4A352 | 0.46508 | 0.01788 | -1.10443 |
| A0A1S3YUR6 | 0.46904 | 0.03339 | -1.09221 |
| A0A1S4BS25 | 0.47102 | 0.01996 | -1.08611 |
| A0A1S3ZLJ6 | 0.47346 | 0.00914 | -1.07867 |
| Q6IVK8 | 0.47411 | 0.01188 | -1.07670 |
| A0A1S3ZRI7 | 0.47426 | 0.03509 | -1.07623 |
| A0A1S4C6R2 | 0.47455 | 0.01787 | -1.07535 |
| A0A1S4B3K4 | 0.48139 | 0.00226 | -1.05469 |
| A0A1S4DQ09 | 0.48364 | 0.03323 | -1.04799 |
| Q5MA39 | 0.48406 | 0.01175 | -1.04673 |
| A0A1S3X9C7 | 0.48772 | 0.00394 | -1.03586 |
| A0A1S4DFX6 | 0.49037 | 0.00402 | -1.02804 |
| A0A1S4D3D0 | 0.49499 | 0.03359 | -1.01452 |
| A0A1S3XCF6 | 0.49769 | 0.00270 | -1.00665 |
| A0A1S4D9D0 | 0.49849 | 0.02572 | -1.00435 |
| A0A1S3XRL0 | 0.49857 | 0.03841 | -1.00412 |
| A0A1S4D6D8 | 0.49882 | 0.00030 | -1.00339 |
| A0A1S4DID7 | 0.49889 | 0.02328 | -1.00320 |
| Q5MA53 | 0.50049 | 0.02791 | -0.99858 |
| A0A1S4BJD7 | 0.50201 | 0.02952 | -0.99420 |
| A0A1S4D2B8 | 0.50436 | 0.00685 | -0.98745 |
| A0A1S4BAJ5 | 0.50756 | 0.00704 | -0.97833 |
| A0A1S3YJ21 | 0.50924 | 0.01151 | -0.97358 |
| A0A1S4AYU6 | 0.51181 | 0.01565 | -0.96632 |
| A0A1S3YA98 | 0.51360 | 0.02263 | -0.96127 |
| A0A1S3YRS7 | 0.51402 | 0.01649 | -0.96010 |
| A0A1S4AX14 | 0.51582 | 0.02938 | -0.95506 |
| A0A1S4C8C0 | 0.51885 | 0.00382 | -0.94659 |
| Q5ZQU1 | 0.51935 | 0.00160 | -0.94519 |
| A0A1S3XKJ8 | 0.52049 | 0.00488 | -0.94206 |
| A0A1S4AUK5 | 0.52522 | 0.03358 | -0.92899 |
| A0A1S4DR24 | 0.52566 | 0.02827 | -0.92778 |
| A0A1S4A5C7 | 0.52652 | 0.00870 | -0.92543 |
| Q8W1L4 | 0.52971 | 0.04343 | -0.91670 |
| A0A1S4A4Y8 | 0.53475 | 0.03380 | -0.90306 |
| A0A1S3Z4G5 | 0.53521 | 0.00689 | -0.90180 |
| A0A1S3XL08 | 0.53540 | 0.03253 | -0.90129 |
| A0A1S4DQ45 | 0.53636 | 0.00113 | -0.89871 |
| A0A1S4CBJ6 | 0.53781 | 0.00024 | -0.89482 |
| A0A1S3ZTW1 | 0.53911 | 0.03842 | -0.89133 |
| A0A1S4CLJ3 | 0.54017 | 0.02297 | -0.88849 |
| A0A1S3ZYZ4 | 0.54043 | 0.03889 | -0.88780 |
| Q5M9U6 | 0.54053 | 0.02408 | -0.88755 |
| A0A1S3XDQ0 | 0.54087 | 0.02383 | -0.88663 |
| A0A1S3ZR63 | 0.54243 | 0.01615 | -0.88248 |
| A0A1S4D6R7 | 0.54243 | 0.03197 | -0.88247 |
| A0A1S4A2T4 | 0.54373 | 0.02673 | -0.87902 |
| A0A1S4D274 | 0.54401 | 0.01962 | -0.87830 |
| A0A1S4A5G5 | 0.54670 | 0.02034 | -0.87116 |
| A0A1S3ZQB4 | 0.55009 | 0.04015 | -0.86226 |
| A0A1S4BKH1 | 0.55171 | 0.00499 | -0.85800 |
| A0A1S4CUG0 | 0.55286 | 0.02180 | -0.85499 |
| A0A1S4A731 | 0.55287 | 0.01318 | -0.85498 |
| A0A1S3XMI2 | 0.55390 | 0.01131 | -0.85228 |
| A0A1S3X312 | 0.55457 | 0.00073 | -0.85054 |
| A0A1S3Y350 | 0.55613 | 0.02119 | -0.84649 |
| A0A1S4AKF4 | 0.55698 | 0.01317 | -0.84430 |
| A0A1S3YVD9 | 0.55704 | 0.03147 | -0.84413 |
| A0A1S4BW53 | 0.55817 | 0.02880 | -0.84121 |
| A0A1S3XSM4 | 0.55878 | 0.02317 | -0.83965 |
| A0A1S3XA41 | 0.56279 | 0.02116 | -0.82933 |
| A0A1S3YSU7 | 0.56288 | 0.00613 | -0.82908 |
| A0A1S4CKZ7 | 0.56416 | 0.02271 | -0.82580 |
| A0A1S3YBM8 | 0.56431 | 0.04538 | -0.82543 |
| A0A1S3YKX4 | 0.56541 | 0.04826 | -0.82262 |
| A0A1S3ZZ18 | 0.56674 | 0.03466 | -0.81922 |
| A0A1S3WXX8 | 0.56728 | 0.04279 | -0.81786 |
| A0A1S3ZSP9 | 0.56916 | 0.02577 | -0.81307 |
| A0A1S4DQ46 | 0.57160 | 0.02683 | -0.80692 |
| A0A1S4AFF1 | 0.57318 | 0.02574 | -0.80294 |
| A0A1S4D9R6 | 0.57750 | 0.00672 | -0.79209 |
| A0A1S4B484 | 0.57818 | 0.00626 | -0.79039 |
| A0A1S4CAK0 | 0.57968 | 0.04618 | -0.78666 |
| A0A1S4AM78 | 0.58095 | 0.02186 | -0.78349 |
| A0A1S3XMX4 | 0.58606 | 0.01064 | -0.77087 |
| A0A1S3X0I8 | 0.58621 | 0.00641 | -0.77051 |
| A0A1S4BXK4 | 0.58751 | 0.03059 | -0.76730 |
| A0A1S3Y4G2 | 0.58766 | 0.01302 | -0.76693 |
| A0A1S4A0W1 | 0.59080 | 0.00359 | -0.75924 |
| A0A1S3X094 | 0.59099 | 0.00130 | -0.75878 |
| A0A1S4D6P6 | 0.59189 | 0.00241 | -0.75659 |
| A0A140G1X4 | 0.59425 | 0.03301 | -0.75086 |
| A0A1S4AX76 | 0.59548 | 0.02987 | -0.74786 |
| A0A1S4CN23 | 0.59619 | 0.04314 | -0.74614 |
| A0A077D9R7 | 0.59788 | 0.04559 | -0.74207 |
| A0A1S3WYS4 | 0.59875 | 0.00050 | -0.73996 |
| A0A1S3YNN4 | 0.59875 | 0.00079 | -0.73996 |
| A0A1S4B620 | 0.59880 | 0.01302 | -0.73985 |
| A0A1S4AAJ2 | 0.60250 | 0.02879 | -0.73096 |
| A1XEK4 | 0.60383 | 0.03481 | -0.72779 |
| A0A1S4D713 | 0.60523 | 0.00012 | -0.72444 |
| A0A1S4ANS2 | 0.605808 | 0.00857 | -0.72307 |
| A0A1S4AC59 | 0.60827 | 0.00085 | -0.71721 |
| A0A1S4CEE8 | 0.60882 | 0.02216 | -0.71590 |
| A0A1S3YPA6 | 0.60916 | 0.01267 | -0.71510 |
| A0A1S4AUA5 | 0.60928 | 0.01217 | -0.71481 |
| A0A1S3YTX6 | 0.61304 | 0.00266 | -0.70593 |
| Q43576 | 0.61415 | 0.01043 | -0.70332 |
| A0A1S4CLA1 | 0.61611 | 0.00316 | -0.69874 |
| A0A1S3Y2B9 | 0.61618 | 0.03016 | -0.69857 |
| A0A1S3Y2C2 | 0.61624 | 0.01909 | -0.69844 |
| A0A1S4DQ26 | 0.61720 | 0.04711 | -0.69617 |
| A0A1S3XEE2 | 0.61735 | 0.04939 | -0.69584 |
| A0A1S4AWG0 | 0.61760 | 0.00544 | -0.69525 |
| A0A1S3ZPC7 | 0.61908 | 0.01457 | -0.69180 |
| Q5M9Z2 | 0.61928 | 0.02444 | -0.69133 |
| A0A0C4FST6 | 0.61934 | 0.03383 | -0.69119 |
| A0A1S4A0H6 | 0.62129 | 0.00611 | -0.68666 |
| A0A1S4D1P6 | 0.62191 | 0.04695 | -0.68521 |
| A0A1S4ACM1 | 0.62229 | 0.02227 | -0.68434 |
| A0A1S4BZQ6 | 0.62453 | 0.01543 | -0.67914 |
| A0A1S4BPV3 | 0.62514 | 0.01248 | -0.67774 |
| Q5XMB8 | 0.63075 | 0.00406 | -0.66485 |
| A0A1S4A451 | 0.63085 | 0.03361 | -0.66463 |
| A0A1S3Y6E5 | 0.63133 | 0.00017 | -0.66352 |
| A0A1S3ZDC5 | 0.63193 | 0.00081 | -0.66214 |
| A0A1S3ZH94 | 0.63278 | 0.01419 | -0.66022 |
| A0A1S3XIZ9 | 0.63483 | 0.02410 | -0.65554 |
| A0A1S3ZMF4 | 0.63577 | 0.00059 | -0.65340 |
| A0A1S3Y1L8 | 0.63676 | 0.02755 | -0.65118 |
| A0A1S3ZM06 | 0.63821 | 0.01049 | -0.64789 |
| A0A1S4C7G6 | 0.64040 | 0.02727 | -0.64294 |
| A0A1S4ABB9 | 0.64320 | 0.03718 | -0.63665 |
| A0A1S3YW36 | 0.64479 | 0.02181 | -0.63308 |
| A0A1S3YFR7 | 0.64609 | 0.02741 | -0.63018 |
| A0A1S3YX82 | 0.64927 | 0.00158 | -0.62310 |
| A0A1S3XIH5 | 0.64929 | 0.00290 | -0.62305 |
| A0A1S4CR50 | 0.65002 | 0.01322 | -0.62144 |
| A0A068JDA3 | 0.65048 | 0.01318 | -0.62042 |
| A0A1S3ZDG3 | 0.65146 | 0.04927 | -0.61825 |
| A0A1S4A7Q4 | 0.65225 | 0.00061 | -0.61648 |
| A0A1S3Y175 | 0.65361 | 0.01106 | -0.61350 |
| A0A1S3XP23 | 0.65481 | 0.03749 | -0.61085 |
| A0A1S4AC43 | 0.65509 | 0.00405 | -0.61023 |
| A0A1S3WY23 | 0.65557 | 0.04565 | -0.60916 |
| A0A1S4ASP0 | 0.65576 | 0.00717 | -0.60875 |
| A0A1S4CX79 | 0.65718 | 0.03654 | -0.60562 |
| A0A1S3Y1G1 | 0.65827 | 0.03880 | -0.60324 |
| A0A1S4ADF8 | 0.65940 | 0.04019 | -0.60076 |
| A0A1S3YE46 | 0.66234 | 0.02272 | -0.59435 |
| A0A1S4DJY8 | 0.66502 | 0.02876 | -0.58851 |
| A0A1S4DLK6 | 0.66526 | 0.00121 | -0.58801 |
| A0A1S4CV55 | 0.66647 | 0.00489 | -0.58537 |
| A0A1S4B0Q0 | 1.50039 | 0.01251 | 0.58534 |
| A0A1S3XCH3 | 1.50052 | 0.02095 | 0.58547 |
| A0A1S3XFW0 | 1.50058 | 0.01067 | 0.58552 |
| A0A1S4AFF5 | 1.50181 | 0.01120 | 0.58671 |
| A0A1S4D7Z3 | 1.50198 | 0.00666 | 0.58686 |
| A0A1S4CST9 | 1.50290 | 0.00811 | 0.58774 |
| A0A1S3YQZ1 | 1.50312 | 0.00215 | 0.58796 |
| Q9SDQ5 | 1.50408 | 0.00086 | 0.58888 |
| A0A1S3YVT7 | 1.50528 | 0.03315 | 0.59003 |
| A0A1S4BA26 | 1.50539 | 0.03026 | 0.59014 |
| A0A1S4DJI1 | 1.50577 | 0.01569 | 0.59050 |
| A0A1S4DQ00 | 1.50585 | 0.01266 | 0.59058 |
| A0A1S4AIX7 | 1.50639 | 0.00604 | 0.59109 |
| Q5DKU6 | 1.50643 | 0.04439 | 0.59114 |
| A0A1S3YSI9 | 1.50654 | 0.00026 | 0.59124 |
| A0A1S4CBR5 | 1.50670 | 0.04466 | 0.59140 |
| A0A1S4AFR6 | 1.50679 | 0.01227 | 0.59148 |
| A0A1S3WYI2 | 1.50703 | 0.04085 | 0.59171 |
| A0A1S4A1M9 | 1.50758 | 0.03196 | 0.59224 |
| A0A1S3Z1E4 | 1.50898 | 0.00467 | 0.59358 |
| A0A1S3ZFX7 | 1.51143 | 0.04209 | 0.59591 |
| A0A1S4D4J1 | 1.51171 | 0.03680 | 0.59618 |
| A0A1S3Y8I0 | 1.51489 | 0.01503 | 0.59921 |
| A0A1S4C6E8 | 1.51494 | 0.00188 | 0.59926 |
| A0A1S3X3R4 | 1.51703 | 0.04183 | 0.60125 |
| A0A1S4BJ90 | 1.51797 | 0.00492 | 0.60214 |
| A0A1S3YEW3 | 1.51847 | 0.04451 | 0.60262 |
| A0A1S3YJV1 | 1.51853 | 0.01234 | 0.60268 |
| A0A1S3Z898 | 1.51881 | 0.02565 | 0.60294 |
| A0A1S3YQC5 | 1.51975 | 0.00815 | 0.60384 |
| A0A1S4ARZ1 | 1.52090 | 0.04866 | 0.60493 |
| A0A1S3YJI3 | 1.52329 | 0.00335 | 0.60719 |
| A0A1S4API1 | 1.52408 | 0.00638 | 0.60794 |
| A0A1S3X828 | 1.52486 | 0.00654 | 0.60868 |
| A0A1S4AWM3 | 1.52706 | 0.02087 | 0.61075 |
| A0A1S4AQH4 | 1.52761 | 0.04970 | 0.61127 |
| A0A1S3ZC53 | 1.52978 | 0.01694 | 0.61333 |
| Q8H6P9 | 1.52996 | 0.02320 | 0.61350 |
| A0A1S4B5J6 | 1.53044 | 0.04765 | 0.61395 |
| A0A1S3ZVC8 | 1.53138 | 0.00261 | 0.61484 |
| A0A1S4B0S5 | 1.53330 | 0.01325 | 0.61664 |
| A0A1S3XPR7 | 1.53334 | 0.00326 | 0.61667 |
| A0A076L1Y1 | 1.53596 | 0.00411 | 0.61914 |
| A0A1S3ZDM8 | 1.53755 | 0.03884 | 0.62063 |
| A0A1S4D973 | 1.53764 | 0.02300 | 0.62072 |
| A0A1S4AHY5 | 1.53774 | 0.01527 | 0.62081 |
| A0A1S4A0E0 | 1.53855 | 0.00136 | 0.62157 |
| A0A1S4B6Y5 | 1.53911 | 0.00332 | 0.62209 |
| A0A1S3YX09 | 1.54132 | 0.00484 | 0.62417 |
| A0A1S4CK90 | 1.54254 | 0.03111 | 0.62530 |
| A0A1S4CSI9 | 1.54272 | 0.00987 | 0.62548 |
| A0A1S3Y8G8 | 1.54279 | 0.03615 | 0.62555 |
| A0A1S3ZE10 | 1.54327 | 0.02900 | 0.62599 |
| A0A1S4CQQ9 | 1.54388 | 0.00397 | 0.62656 |
| A0A1S3ZBV2 | 1.54452 | 0.03424 | 0.62716 |
| A0A140G1S3 | 1.54510 | 0.00851 | 0.62770 |
| A0A1S3YT47 | 1.54581 | 0.00723 | 0.62836 |
| A0A1S4D6C0 | 1.54697 | 0.01079 | 0.62944 |
| A0A1S3YQ99 | 1.54773 | 0.00819 | 0.63016 |
| A0A1S3WYR8 | 1.54882 | 0.01993 | 0.63117 |
| A0A1S4CM51 | 1.54890 | 0.02980 | 0.63124 |
| A0A1S4DE74 | 1.54900 | 0.02409 | 0.63134 |
| A0A1S4AXA8 | 1.54933 | 0.03258 | 0.63164 |
| A0A1S4DJK0 | 1.55024 | 0.03227 | 0.63249 |
| A0A1S4CC61 | 1.55122 | 0.02940 | 0.63340 |
| A0A1S4D2L1 | 1.55124 | 0.01144 | 0.63342 |
| A0A1S3YED7 | 1.55134 | 0.00687 | 0.63352 |
| A0A1S3YTB9 | 1.55145 | 0.00999 | 0.63361 |
| A0A1S4CB55 | 1.55278 | 0.01307 | 0.63485 |
| A0A1S3YBJ9 | 1.55426 | 0.03259 | 0.63623 |
| A0A1S3ZVH3 | 1.55435 | 0.01191 | 0.63631 |
| A0A1S4DFZ6 | 1.55450 | 0.02361 | 0.63645 |
| A0A1S3ZRF6 | 1.55548 | 0.00098 | 0.63736 |
| A0A1S4CGB1 | 1.55616 | 0.00085 | 0.63799 |
| A0A1S3XMH2 | 1.55664 | 0.04386 | 0.63844 |
| A0A1S4AW90 | 1.55692 | 0.04662 | 0.63869 |
| A0A1S3ZPB2 | 1.55764 | 0.00338 | 0.63936 |
| A0A1S4DGA3 | 1.55830 | 0.00659 | 0.63997 |
| A0A1S4A2X7 | 1.56326 | 0.03093 | 0.64456 |
| A0A1S4DHJ8 | 1.56454 | 0.01077 | 0.64574 |
| A0A1S3Z9L1 | 1.56521 | 0.03185 | 0.64635 |
| Q8SBC6 | 1.56544 | 0.02069 | 0.64657 |
| A0A1S4B7U8 | 1.56545 | 0.02447 | 0.64657 |
| A0A1S4CVQ8 | 1.56571 | 0.04168 | 0.64682 |
| A0A1S4D963 | 1.56583 | 0.02826 | 0.64693 |
| A0A1S4D3R4 | 1.56727 | 0.01541 | 0.64825 |
| A0A1S4BLM6 | 1.56761 | 0.02229 | 0.64856 |
| A0A1S3Y0W1 | 1.56836 | 0.03935 | 0.64926 |
| A0A1S4AFU8 | 1.56848 | 0.03874 | 0.64936 |
| A0A1S4B9X1 | 1.57046 | 0.01451 | 0.65119 |
| A0A1S4DMW4 | 1.57166 | 0.01752 | 0.65229 |
| A0A1S3YV51 | 1.57171 | 0.04120 | 0.65233 |
| A0A1S3ZB94 | 1.57245 | 0.02587 | 0.65301 |
| A0A1S4AUN0 | 1.57252 | 0.00814 | 0.65308 |
| A0A1S3ZA22 | 1.57324 | 0.01862 | 0.65374 |
| Q3LAG6 | 1.57330 | 0.03762 | 0.65380 |
| A0A1S4A837 | 1.57336 | 0.00562 | 0.65385 |
| A0A1S3XAH3 | 1.57339 | 0.00589 | 0.65388 |
| A0A1S3ZQ67 | 1.57364 | 0.00552 | 0.65410 |
| A0A1S4BP94 | 1.57465 | 0.00382 | 0.65504 |
| A0A1S3ZY65 | 1.57586 | 0.02698 | 0.65614 |
| A0A1S4CGS1 | 1.57605 | 0.01087 | 0.65632 |
| A0A140G1R1 | 1.57620 | 0.00720 | 0.65645 |
| A0A1S4CK55 | 1.57732 | 0.01005 | 0.65748 |
| A0A1S4B192 | 1.57811 | 0.03099 | 0.65820 |
| A0A1S3XGJ0 | 1.57947 | 0.00299 | 0.65944 |
| A0A1S4ACH2 | 1.58085 | 0.01542 | 0.66070 |
| A0A140G1S1 | 1.58107 | 0.01901 | 0.66091 |
| A0A1S4DJQ0 | 1.58195 | 0.01188 | 0.66171 |
| A0A1S4DFE3 | 1.58253 | 0.03343 | 0.66223 |
| A0A1S4B7Q9 | 1.58284 | 0.02447 | 0.66252 |
| A0A1S3XM68 | 1.58315 | 0.02783 | 0.66279 |
| A0A1S4BLP8 | 1.58366 | 0.02568 | 0.66326 |
| A0A1S3X0U3 | 1.58534 | 0.02135 | 0.66479 |
| A0A1S4CIH1 | 1.58592 | 0.03008 | 0.66532 |
| A0A1S4B2Q9 | 1.58616 | 0.04670 | 0.66554 |
| A0A1S3ZWP0 | 1.58639 | 0.00470 | 0.66575 |
| A0A1S3YUN2 | 1.58648 | 0.01646 | 0.66583 |
| A0A1S4CF99 | 1.58691 | 0.00334 | 0.66622 |
| A0A1S3ZJS0 | 1.58789 | 0.02843 | 0.66711 |
| A0A1S3XFX9 | 1.58824 | 0.01356 | 0.66743 |
| A0A1S3Y9G7 | 1.58872 | 0.00268 | 0.66786 |
| A0A1S4CZ48 | 1.58973 | 0.00336 | 0.66878 |
| A0A1S4ARW4 | 1.58995 | 0.03269 | 0.66898 |
| A0A1S3XHZ3 | 1.59012 | 0.01406 | 0.66914 |
| A0A1S4CKD3 | 1.59240 | 0.04088 | 0.67120 |
| A0A1S3Y7M2 | 1.59262 | 0.01499 | 0.67140 |
| A0A1S4CKZ6 | 1.59287 | 0.01878 | 0.67163 |
| A0A1S3ZYF1 | 1.59506 | 0.02230 | 0.67361 |
| A0A1S4AF88 | 1.59537 | 0.01488 | 0.67389 |
| A0A1S4DDW9 | 1.59805 | 0.00557 | 0.67631 |
| A0A1S4BAT9 | 1.59924 | 0.00548 | 0.67738 |
| A0A1S4BRV8 | 1.59960 | 0.02156 | 0.67771 |
| A0A1S3ZBU9 | 1.60054 | 0.01283 | 0.67856 |
| A0A1S4DRW2 | 1.60063 | 0.01770 | 0.67864 |
| A0A1S4D6L6 | 1.60258 | 0.01777 | 0.68040 |
| A0A1S4DKM6 | 1.60302 | 0.01644 | 0.68079 |
| A0A1S4DQV4 | 1.60327 | 0.01705 | 0.68102 |
| A0A1S3YS78 | 1.60414 | 0.02491 | 0.68180 |
| A0A1S4B3X4 | 1.60549 | 0.03827 | 0.68302 |
| A0A1S4BW60 | 1.60685 | 0.00523 | 0.68424 |
| A0A1S3YXP2 | 1.60792 | 0.00167 | 0.68520 |
| A0A1S3ZLW1 | 1.60877 | 0.04848 | 0.68596 |
| A0A1S3X387 | 1.60928 | 0.00480 | 0.68642 |
| A0A1S3ZW46 | 1.60989 | 0.02137 | 0.68696 |
| A0A1S4C9K9 | 1.61031 | 0.01764 | 0.68734 |
| A0A1S3YPD2 | 1.61073 | 0.02105 | 0.68772 |
| A0A1S4C9W7 | 1.61079 | 0.04623 | 0.68777 |
| A0A1S4CDV5 | 1.61440 | 0.02202 | 0.69100 |
| A0A1S4BLD8 | 1.61671 | 0.00619 | 0.69306 |
| A0A1S3ZTX1 | 1.61855 | 0.00487 | 0.69470 |
| A0A1S3ZI30 | 1.61966 | 0.00243 | 0.69569 |
| A0A1S3YHS3 | 1.62098 | 0.01367 | 0.69687 |
| A0A1S4D980 | 1.62106 | 0.03988 | 0.69693 |
| A0A1S4ATD5 | 1.62222 | 0.03376 | 0.69796 |
| A0A1S4CZH1 | 1.62269 | 0.02305 | 0.69838 |
| A0A1S4BGM4 | 1.62303 | 0.00072 | 0.69869 |
| A0A9R6 | 1.62321 | 0.04559 | 0.69885 |
| A0A1S3ZFF9 | 1.62384 | 0.01098 | 0.69941 |
| A0A1S4D649 | 1.62421 | 0.00923 | 0.69973 |
| A0A1S4APL7 | 1.62442 | 0.01746 | 0.69993 |
| A0A1S4DJC2 | 1.62722 | 0.01850 | 0.70241 |
| A0A1S4AZ56 | 1.62808 | 0.00062 | 0.70317 |
| A0A1S4AM69 | 1.62862 | 0.03538 | 0.70365 |
| A0A1S3ZT94 | 1.62883 | 0.01728 | 0.70384 |
| A0A1S4B3V4 | 1.63037 | 0.03454 | 0.70520 |
| A0A1S4ANZ2 | 1.63237 | 0.02530 | 0.70697 |
| A0A1S3ZMK4 | 1.63368 | 0.00862 | 0.70812 |
| A0A1S3XRM3 | 1.63621 | 0.01860 | 0.71035 |
| A0A1S3Y611 | 1.63665 | 0.01564 | 0.71075 |
| A0A1S3WXB3 | 1.63692 | 0.02790 | 0.71098 |
| A0A1S4CEG6 | 1.63820 | 0.00504 | 0.71211 |
| A0A1S4CNI7 | 1.63961 | 0.03421 | 0.71335 |
| A0A1S3XFZ2 | 1.63973 | 0.00506 | 0.71346 |
| A0A1S4AME4 | 1.64186 | 0.00601 | 0.71533 |
| A0A1S3ZIV1 | 1.64609 | 0.03200 | 0.71904 |
| A0A075F933 | 1.64622 | 0.03455 | 0.71916 |
| A0A1S3YLE9 | 1.64740 | 0.01407 | 0.72019 |
| A0A1S3ZA20 | 1.64865 | 0.00056 | 0.72129 |
| A0A1S4CTN4 | 1.65101 | 0.00950 | 0.72334 |
| A0A1S4DLE6 | 1.65195 | 0.01646 | 0.72417 |
| A0A1S4D3U7 | 1.65201 | 0.02272 | 0.72422 |
| A0A1S4ACK9 | 1.65214 | 0.03575 | 0.72434 |
| A0A1S3ZPM0 | 1.65299 | 0.04703 | 0.72508 |
| A0A1S4A9Y0 | 1.65307 | 0.00809 | 0.72515 |
| A0A1S3XT44 | 1.65502 | 0.03696 | 0.72685 |
| A0A1S4C9Q1 | 1.65527 | 0.01582 | 0.72706 |
| A0A1S4APN5 | 1.65883 | 0.04098 | 0.73016 |
| A0A1S4A0F9 | 1.65950 | 0.04700 | 0.73075 |
| A0A1S4CAC1 | 1.66092 | 0.04977 | 0.73198 |
| A0A1S3ZJC9 | 1.66155 | 0.00167 | 0.73253 |
| A0A1S4AKD3 | 1.66551 | 0.00561 | 0.73596 |
| D4P3S0 | 1.66554 | 0.03456 | 0.73599 |
| A0A1S3X463 | 1.66605 | 0.01969 | 0.73643 |
| A0A1S4D1X6 | 1.66657 | 0.01018 | 0.73688 |
| A0A1S3YLB2 | 1.66830 | 0.00902 | 0.73838 |
| A0A1S4AQL1 | 1.67006 | 0.02603 | 0.73990 |
| A0A1S4ALW6 | 1.67142 | 0.03699 | 0.74107 |
| A0A1S3YVM0 | 1.67170 | 0.03740 | 0.74131 |
| A0A1S3XHU5 | 1.67187 | 0.01422 | 0.74146 |
| A0A1S4CH99 | 1.67444 | 0.00138 | 0.74368 |
| A0A1S4DGP1 | 1.67618 | 0.03418 | 0.74518 |
| A0A1S4CTH1 | 1.67786 | 0.03973 | 0.74662 |
| A0A1S3XJD7 | 1.67911 | 0.00827 | 0.74770 |
| A0A1S3YUU3 | 1.67920 | 0.03631 | 0.74777 |
| A0A1S4AWH0 | 1.68342 | 0.00830 | 0.75140 |
| A0A1S3Y1X0 | 1.68343 | 0.03789 | 0.75141 |
| A0A1S3YCS0 | 1.68350 | 0.00945 | 0.75146 |
| A0A1S4BQD4 | 1.68409 | 0.02391 | 0.75197 |
| A0A1S4A792 | 1.68631 | 0.04061 | 0.75387 |
| A0A1S4CS74 | 1.68861 | 0.00400 | 0.75583 |
| A0A1S4DNL1 | 1.68898 | 0.02124 | 0.75616 |
| A0A1S4CVG9 | 1.68899 | 0.02794 | 0.75616 |
| A0A1S4BSS6 | 1.68998 | 0.01216 | 0.75700 |
| A0A1S3ZP17 | 1.69033 | 0.03361 | 0.75730 |
| A0A1S3YSD3 | 1.69059 | 0.02563 | 0.75753 |
| A0A1S3ZI39 | 1.69428 | 0.04666 | 0.76067 |
| A0A1S4A6S1 | 1.69497 | 0.04056 | 0.76126 |
| A0A1S3XYU8 | 1.69592 | 0.00171 | 0.76207 |
| D3W9H9 | 1.69864 | 0.02574 | 0.76438 |
| A0A1S4AG60 | 1.70112 | 0.04500 | 0.76649 |
| A0A1S3ZAZ0 | 1.70200 | 0.02581 | 0.76723 |
| A0A1S3X5Z7 | 1.70514 | 0.04388 | 0.76989 |
| A0A1S4BJ07 | 1.70756 | 0.00021 | 0.77193 |
| A0A1S3Z8A6 | 1.70881 | 0.00380 | 0.77300 |
| A0A1S4BKP9 | 1.70942 | 0.00045 | 0.77351 |
| A0A1S3WYW6 | 1.71069 | 0.04342 | 0.77457 |
| A0A1S4AYZ4 | 1.71093 | 0.02445 | 0.77478 |
| A0A1S4BQT4 | 1.71176 | 0.03120 | 0.77548 |
| A0A1S3ZGU9 | 1.71508 | 0.00699 | 0.77828 |
| A0A1S4A3L7 | 1.71574 | 0.03074 | 0.77883 |
| A0A1S4D9D2 | 1.72136 | 0.02019 | 0.78355 |
| A0A1S3Z8C6 | 1.72234 | 0.03320 | 0.78437 |
| A0A1S3ZPU0 | 1.72445 | 0.02528 | 0.78614 |
| A0A1S3XV30 | 1.72570 | 0.01327 | 0.78718 |
| A0A1S3ZA83 | 1.72608 | 0.01052 | 0.78749 |
| A0A1S3YEC4 | 1.72786 | 0.03918 | 0.78899 |
| A0A1S3Z496 | 1.72792 | 0.02804 | 0.78904 |
| A0A1S4A1Q7 | 1.72956 | 0.00096 | 0.79041 |
| A0A1S3ZUR7 | 1.73094 | 0.00057 | 0.79156 |
| A0A1S3WXL4 | 1.73122 | 0.03692 | 0.79179 |
| A0A075M5G1 | 1.73390 | 0.04768 | 0.79402 |
| A0A1S3WYW5 | 1.73429 | 0.01101 | 0.79434 |
| A0A1S3X4S6 | 1.73487 | 0.01759 | 0.79483 |
| A0A1S4CJV0 | 1.73568 | 0.00817 | 0.79550 |
| A0A1S4AEA0 | 1.73577 | 0.02821 | 0.79557 |
| A0A1S4BYM4 | 1.73597 | 0.00591 | 0.79574 |
| A0A076L1Y6 | 1.73612 | 0.00203 | 0.79587 |
| A0A1S3YXD0 | 1.73713 | 0.01479 | 0.79670 |
| A0A1S3YAU5 | 1.74067 | 0.00540 | 0.79964 |
| A0A1S3ZNU8 | 1.74086 | 0.04086 | 0.79980 |
| A0A1S4AUT7 | 1.74094 | 0.01167 | 0.79987 |
| A0A1S3YDQ8 | 1.74163 | 0.04082 | 0.80044 |
| A0A1S3XDA5 | 1.74405 | 0.02032 | 0.80244 |
| A0A1S3XBT8 | 1.74618 | 0.00173 | 0.80420 |
| A0A1S4AXT2 | 1.74723 | 0.00679 | 0.80507 |
| A0A1S3XTD4 | 1.74794 | 0.02147 | 0.80565 |
| A0A1S3XHX0 | 1.74832 | 0.02733 | 0.80597 |
| A0A1S4BZ26 | 1.74859 | 0.03308 | 0.80619 |
| A0A1S4AW82 | 1.75209 | 0.02887 | 0.80908 |
| A0A1S4C067 | 1.75260 | 0.02803 | 0.80949 |
| A0A1S4CN68 | 1.75426 | 0.00829 | 0.81086 |
| A0A1S3ZBB0 | 1.75490 | 0.04210 | 0.81139 |
| A0A1S4AAZ7 | 1.75726 | 0.04526 | 0.81332 |
| A0A1S4BP84 | 1.75874 | 0.00845 | 0.81454 |
| O24163 | 1.75941 | 0.04516 | 0.81509 |
| A0A1S4A6K1 | 1.76007 | 0.03779 | 0.81564 |
| A0A1S4D403 | 1.76231 | 0.00036 | 0.81747 |
| A0A1S3Z9K1 | 1.76246 | 0.01257 | 0.81759 |
| A0A1S3XLH4 | 1.76471 | 0.01140 | 0.81943 |
| A0A1S3YY31 | 1.76503 | 0.00656 | 0.81969 |
| A0A1S3ZLD2 | 1.76658 | 0.00819 | 0.82096 |
| A0A1S3XRQ5 | 1.76680 | 0.02083 | 0.82114 |
| A0A1S3Z7F9 | 1.76815 | 0.04637 | 0.82224 |
| A0A1S4BG47 | 1.76967 | 0.04576 | 0.82348 |
| A0A1S4C2M0 | 1.77131 | 0.01994 | 0.82482 |
| A0A1S4CVG6 | 1.77184 | 0.04645 | 0.82525 |
| A0A1S4BTV5 | 1.77299 | 0.00064 | 0.82618 |
| A0A1S4CSS8 | 1.77567 | 0.00525 | 0.82837 |
| A0A1S4AEZ8 | 1.77664 | 0.00254 | 0.82915 |
| A0A1S4C4S2 | 1.77795 | 0.02926 | 0.83021 |
| A0A1S4DLK0 | 1.77801 | 0.02255 | 0.83027 |
| A0A1S3XJX0 | 1.78019 | 0.00868 | 0.83203 |
| A0A1S3X7W8 | 1.78165 | 0.04252 | 0.83321 |
| A0A1S4CU12 | 1.78474 | 0.01055 | 0.83571 |
| A0A1S4AE99 | 1.78567 | 0.00206 | 0.83647 |
| A0A1S4BNE9 | 1.79022 | 0.00969 | 0.84013 |
| A0A1S4DL73 | 1.79205 | 0.03850 | 0.84161 |
| A0A1S3ZN47 | 1.79221 | 0.02992 | 0.84174 |
| A0A1S3YIQ8 | 1.79270 | 0.02573 | 0.84213 |
| A0A1S4B2P7 | 1.79414 | 0.03258 | 0.84329 |
| A0A1S4AL80 | 1.79442 | 0.02736 | 0.84351 |
| A0A1S4DAQ0 | 1.79496 | 0.03665 | 0.84395 |
| A0A1S3YCF2 | 1.79657 | 0.00748 | 0.84524 |
| A0A1S3XNK3 | 1.79861 | 0.03633 | 0.84688 |
| A0A1S4A1M7 | 1.80124 | 0.04939 | 0.84899 |
| A0A1S3Y8B5 | 1.80311 | 0.04458 | 0.85048 |
| A0A1S3YYD8 | 1.80337 | 0.00785 | 0.85070 |
| A0A1S4D5C4 | 1.80356 | 0.02425 | 0.85085 |
| A0A1S3XHL0 | 1.80564 | 0.01296 | 0.85251 |
| A0A1S4BQ99 | 1.80719 | 0.00410 | 0.85375 |
| A0A1S4DE89 | 1.81158 | 0.01223 | 0.85725 |
| A0A1S3XVG9 | 1.81854 | 0.03539 | 0.86278 |
| A0A1S4B348 | 1.81985 | 0.01089 | 0.86382 |
| A0A1S4AG17 | 1.82153 | 0.04293 | 0.86515 |
| A0A1S4CJ66 | 1.82575 | 0.02393 | 0.86849 |
| A0A1S4BS82 | 1.82809 | 0.01018 | 0.87033 |
| A0A1S4BID9 | 1.82973 | 0.03522 | 0.87163 |
| A0A1S4AMB3 | 1.83039 | 0.00907 | 0.87215 |
| A0A1S3XQB3 | 1.83110 | 0.00353 | 0.87271 |
| A0A1S4CIY0 | 1.83462 | 0.03258 | 0.87548 |
| A0A1S3XRF1 | 1.83775 | 0.02531 | 0.87794 |
| A0A1S4AJJ4 | 1.84041 | 0.00263 | 0.88002 |
| Q04065 | 1.84178 | 0.04866 | 0.88110 |
| A0A1S4AKP0 | 1.84902 | 0.02929 | 0.88676 |
| A0A0K0XR61 | 1.85005 | 0.01598 | 0.88757 |
| A0A1S3XYA7 | 1.85145 | 0.00868 | 0.88866 |
| A0A1S4AN14 | 1.85376 | 0.00607 | 0.89046 |
| A0A1S4AUX3 | 1.85746 | 0.00261 | 0.89333 |
| A0A1S3YA92 | 1.86015 | 0.01833 | 0.89542 |
| A0A1S3ZAP9 | 1.86027 | 0.02635 | 0.89551 |
| A0A1S3XPW7 | 1.86080 | 0.00079 | 0.89593 |
| A0A1S4CMH6 | 1.86490 | 0.03074 | 0.89910 |
| A0A1S3YLK8 | 1.86490 | 0.04298 | 0.89910 |
| A0A1S4C687 | 1.86582 | 0.02727 | 0.89981 |
| A0A1S3YU63 | 1.86672 | 0.00473 | 0.90051 |
| A0A1S4C620 | 1.86727 | 0.00760 | 0.90093 |
| A0A1S3ZYY3 | 1.86803 | 0.01171 | 0.90152 |
| A0A1S4B471 | 1.87183 | 0.04056 | 0.90445 |
| A0A1S4C341 | 1.87244 | 0.01865 | 0.90492 |
| Q9M6E7 | 1.87251 | 0.00936 | 0.90497 |
| A0A1S4C081 | 1.87656 | 0.02223 | 0.90809 |
| A0A1S3YN60 | 1.87683 | 0.00804 | 0.90830 |
| A0A1S3Y2E3 | 1.87824 | 0.02950 | 0.90938 |
| A0A1S4AP98 | 1.87892 | 0.00820 | 0.90990 |
| A0A1S4BAA7 | 1.88171 | 0.00575 | 0.91204 |
| A0A1S3Y3J3 | 1.88405 | 0.04307 | 0.91383 |
| A0A1S3ZLR0 | 1.88987 | 0.03429 | 0.91828 |
| A0A1S3ZKZ6 | 1.89155 | 0.01058 | 0.91956 |
| A0A1S3ZFR9 | 1.89458 | 0.00703 | 0.92187 |
| A0A1S3Y7Y6 | 1.90178 | 0.04233 | 0.92735 |
| A0A1S4B5E7 | 1.90733 | 0.00953 | 0.93155 |
| A0A1S3YHZ6 | 1.90778 | 0.00216 | 0.93189 |
| A0A1S4B6D4 | 1.90829 | 0.00691 | 0.93228 |
| A0A1S3YWF6 | 1.90997 | 0.03764 | 0.93355 |
| A0A1S4B899 | 1.91649 | 0.00292 | 0.93846 |
| A0A1S3XVV3 | 1.91722 | 0.02357 | 0.93901 |
| A0A1S4BCH4 | 1.91971 | 0.00099 | 0.94089 |
| A0A1S3ZXK8 | 1.91977 | 0.02427 | 0.94093 |
| A0A1S4A4U2 | 1.92062 | 0.03360 | 0.94157 |
| A0A1S4DFL7 | 1.92192 | 0.02202 | 0.94255 |
| A0A1S3X9W7 | 1.92454 | 0.04217 | 0.94451 |
| D6PZY5 | 1.93000 | 0.00145 | 0.94860 |
| A0A1S3ZFJ3 | 1.93533 | 0.00260 | 0.95258 |
| Q9MUE2 | 1.93815 | 0.01870 | 0.95468 |
| A0A1S3Y006 | 1.93831 | 0.03203 | 0.95480 |
| A0A1S4DD08 | 1.94819 | 0.01021 | 0.96213 |
| A0A1S4CDK3 | 1.94877 | 0.00027 | 0.96256 |
| A0A1S3YFN1 | 1.94970 | 0.00688 | 0.96325 |
| A0A1S4D4V1 | 1.95364 | 0.01223 | 0.96616 |
| A0A1S4A379 | 1.95675 | 0.01373 | 0.96846 |
| A0A1S3ZDN3 | 1.95960 | 0.01571 | 0.97056 |
| A0A1S4DNF7 | 1.96013 | 0.03376 | 0.97095 |
| A0A1S4CVR8 | 1.96280 | 0.02504 | 0.97291 |
| A0A1S3YEF7 | 1.96367 | 0.01748 | 0.97355 |
| A0A1S3Z3N3 | 1.96727 | 0.00375 | 0.97619 |
| A0A1S4D2Y6 | 1.96864 | 0.00037 | 0.97720 |
| A0A1S3ZDR5 | 1.97008 | 0.02276 | 0.97825 |
| A0A1S4AX73 | 1.97044 | 0.03689 | 0.97852 |
| A0A1S4BEA7 | 1.97175 | 0.01254 | 0.97947 |
| O49910 | 1.97501 | 0.00251 | 0.98186 |
| A0A1S3ZTL3 | 1.97715 | 0.04512 | 0.98342 |
| A0A1S3YYF0 | 1.97779 | 0.01009 | 0.98389 |
| A0A1S3XXP1 | 1.97994 | 0.00649 | 0.98546 |
| A0A1S4BPK4 | 1.98085 | 0.03348 | 0.98612 |
| A0A1S3YKV1 | 1.98089 | 0.01458 | 0.98614 |
| A0A1S4C5L7 | 1.99074 | 0.04086 | 0.99331 |
| A0A1S4AMI2 | 1.99093 | 0.00001 | 0.99344 |
| A0A1S3Y3M5 | 1.99532 | 0.00130 | 0.99662 |
| A0A1S4AVE2 | 1.99901 | 0.00282 | 0.99928 |
| A0A1S3XKN4 | 2.00085 | 0.03427 | 1.00061 |
| A0A1S3ZDE0 | 2.00122 | 0.04161 | 1.00088 |
| A0A1S4BNY0 | 2.00394 | 0.03595 | 1.00284 |
| A0A1S4C5L2 | 2.01160 | 0.00288 | 1.00835 |
| A0A1S3ZQL0 | 2.01197 | 0.04604 | 1.00860 |
| A0A1S4DQX2 | 2.01652 | 0.04002 | 1.01187 |
| A0A1S3YRR1 | 2.01919 | 0.00688 | 1.01377 |
| A0A1S4AK69 | 2.02184 | 0.03706 | 1.01567 |
| A0A1S3XAR5 | 2.02296 | 0.00427 | 1.01647 |
| A0A077D849 | 2.02388 | 0.00162 | 1.01713 |
| A0A075EYQ6 | 2.02639 | 0.00050 | 1.01891 |
| A0A1S4BCZ4 | 2.02811 | 0.02527 | 1.02014 |
| A0A1S3Y1D9 | 2.03334 | 0.04719 | 1.02385 |
| A0A1S4C967 | 2.03783 | 0.00350 | 1.02704 |
| A0A1S4BNU0 | 2.03926 | 0.04303 | 1.02804 |
| A0A1S3XC04 | 2.03996 | 0.01119 | 1.02854 |
| A0A1S3WX93 | 2.04166 | 0.02459 | 1.02974 |
| A0A1S3YHI6 | 2.04355 | 0.00079 | 1.03107 |
| A0A1S3YMA4 | 2.04787 | 0.00305 | 1.03412 |
| A0A1S3XYY2 | 2.05925 | 0.00254 | 1.04212 |
| A0A1S3Y880 | 2.06234 | 0.00522 | 1.04428 |
| A0A1S4CCB2 | 2.06327 | 0.02474 | 1.04493 |
| A0A1S4DGZ7 | 2.06615 | 0.02739 | 1.04695 |
| A0A1S4CB73 | 2.06636 | 0.02348 | 1.04709 |
| A0A1S4A364 | 2.06796 | 0.01835 | 1.04821 |
| A0A1S3XHG4 | 2.08373 | 0.00139 | 1.05917 |
| A0A1S3XKY8 | 2.08637 | 0.00183 | 1.06099 |
| A0A1S4BG93 | 2.08940 | 0.04567 | 1.06309 |
| A0A1S3YLT7 | 2.09051 | 0.01566 | 1.06385 |
| A0A0D3L6R2 | 2.09177 | 0.01318 | 1.06472 |
| A0A1S4DG74 | 2.09458 | 0.01921 | 1.06666 |
| A0A1S4BMA4 | 2.10415 | 0.03337 | 1.07324 |
| A0A1S4A1X2 | 2.11151 | 0.00923 | 1.07827 |
| A0A1S4DGJ7 | 2.11206 | 0.03937 | 1.07865 |
| A0A1S3YL10 | 2.12126 | 0.00797 | 1.08492 |
| Q84QE5 | 2.12470 | 0.00455 | 1.08726 |
| A0A1S4AKW7 | 2.12549 | 0.01452 | 1.08780 |
| A0A1S4BF11 | 2.12899 | 0.00250 | 1.09017 |
| A0A1S3Z6C1 | 2.12899 | 0.04224 | 1.09017 |
| A0A1S3Z9I8 | 2.12929 | 0.00426 | 1.09037 |
| A0A1S4CCW1 | 2.13111 | 0.02402 | 1.09160 |
| A0A1S4AG82 | 2.13410 | 0.01754 | 1.09363 |
| A0A1S4A2R3 | 2.14574 | 0.01065 | 1.10147 |
| A0A1S4ARJ1 | 2.16079 | 0.01412 | 1.11156 |
| A0A1S4DKW5 | 2.16312 | 0.01188 | 1.11311 |
| A0A1S3ZX20 | 2.16624 | 0.01339 | 1.11519 |
| A0A1S3ZDY8 | 2.16652 | 0.02713 | 1.11538 |
| A0A1S4CDH7 | 2.16703 | 0.02584 | 1.11572 |
| P09043 | 2.16936 | 0.00195 | 1.11727 |
| A0A0K1CWK2 | 2.16963 | 0.03749 | 1.11745 |
| A0A1S4BI60 | 2.17098 | 0.00368 | 1.11834 |
| D2K7Z2 | 2.17312 | 0.04587 | 1.11977 |
| A0A1S3XM41 | 2.18649 | 0.01300 | 1.12862 |
| A0A1S4AR72 | 2.18753 | 0.00623 | 1.12930 |
| A0A1S4AE69 | 2.18825 | 0.03326 | 1.12977 |
| A0A1S3XS26 | 2.19389 | 0.02699 | 1.13349 |
| A0A1S4AW30 | 2.19592 | 0.04432 | 1.13482 |
| A0A1S3X4K3 | 2.19921 | 0.01185 | 1.13699 |
| A0A1S3XKS0 | 2.20129 | 0.00179 | 1.13835 |
| A0A1S3Z7M7 | 2.20524 | 0.04580 | 1.14094 |
| A0A1S3ZN71 | 2.20644 | 0.04487 | 1.14172 |
| A0A1S3XRS7 | 2.20659 | 0.00183 | 1.14181 |
| A0A1S4CL28 | 2.21252 | 0.00055 | 1.14569 |
| A0A1S4BV37 | 2.21357 | 0.03213 | 1.14637 |
| A0A1S3Y0B9 | 2.23970 | 0.00889 | 1.16331 |
| A0A1S4CHR0 | 2.24050 | 0.02757 | 1.16382 |
| A0A1S3XKV6 | 2.24252 | 0.00637 | 1.16512 |
| A0A1S3XJB3 | 2.24380 | 0.02099 | 1.16594 |
| A0A1S4A939 | 2.25741 | 0.00472 | 1.17466 |
| A0A1S3ZPS2 | 2.25854 | 0.03207 | 1.17539 |
| A0A1S3YYF2 | 2.26137 | 0.01970 | 1.17719 |
| A0A1S3ZVR9 | 2.26329 | 0.01294 | 1.17842 |
| A0A1S3ZCY7 | 2.26944 | 0.00430 | 1.18233 |
| A0A1S3YVC0 | 2.28861 | 0.01942 | 1.19447 |
| A0A1S4BPN0 | 2.29051 | 0.02826 | 1.19567 |
| A0A077LAP2 | 2.30928 | 0.01954 | 1.20744 |
| A0A1S4BSD6 | 2.31079 | 0.00173 | 1.20839 |
| A0A1S3YPR8 | 2.31244 | 0.00240 | 1.20942 |
| A0A1S4A845 | 2.31336 | 0.04714 | 1.20999 |
| A0A1S3ZFD1 | 2.31915 | 0.01563 | 1.21359 |
| A0A076KWG6 | 2.31927 | 0.00136 | 1.21367 |
| A0A1S3X430 | 2.32924 | 0.01222 | 1.21986 |
| A0A1S3XUP9 | 2.33128 | 0.02299 | 1.22112 |
| A0A1S4CUI5 | 2.34407 | 0.01046 | 1.22901 |
| I2G7N7 | 2.35466 | 0.00052 | 1.23551 |
| A0A1S3ZI05 | 2.35827 | 0.01962 | 1.23773 |
| A0A1S3ZBF0 | 2.35873 | 0.01552 | 1.23801 |
| A0A1S3Y6U9 | 2.37817 | 0.00229 | 1.24985 |
| Q1KV12 | 2.37989 | 0.01397 | 1.25089 |
| A0A1S4C1W9 | 2.38352 | 0.00480 | 1.25309 |
| A0A1S3XAV6 | 2.39457 | 0.00456 | 1.25976 |
| A0A1S3XXL6 | 2.40766 | 0.02900 | 1.26763 |
| A0A1S3XUV0 | 2.41058 | 0.02873 | 1.26938 |
| A0A1S3ZZC5 | 2.41670 | 0.01111 | 1.27303 |
| A0A1S4CMV2 | 2.45529 | 0.00001 | 1.29589 |
| A0A1S3XVI5 | 2.48251 | 0.04368 | 1.31179 |
| A0A1S4DNK6 | 2.49875 | 0.00310 | 1.32120 |
| K0I7G7 | 2.51789 | 0.02508 | 1.33221 |
| A0A1S4CT71 | 2.52138 | 0.00669 | 1.33421 |
| A0A1S3X8M2 | 2.52411 | 0.00081 | 1.33577 |
| A0A1S3YX54 | 2.52942 | 0.01394 | 1.33880 |
| A0A1S4B3M0 | 2.53147 | 0.00843 | 1.33997 |
| A0A1S4CAI8 | 2.53336 | 0.02831 | 1.34105 |
| A0A1S3ZXI6 | 2.55965 | 0.03138 | 1.35594 |
| A0A1S4CNK3 | 2.56585 | 0.00156 | 1.35943 |
| A0A1S3XYY7 | 2.61609 | 0.01910 | 1.38741 |
| A0A1S3Y108 | 2.61792 | 0.03731 | 1.38842 |
| A0A1S4AFB0 | 2.62070 | 0.01136 | 1.38995 |
| A0A140G1Q0 | 2.62634 | 0.01215 | 1.39305 |
| A0A1S3X130 | 2.64570 | 0.00450 | 1.40365 |
| A0A1S4DCE5 | 2.64732 | 0.00499 | 1.40453 |
| A0A1S3X3P5 | 2.65775 | 0.00090 | 1.41021 |
| A0A1S4DD78 | 2.66718 | 0.02267 | 1.41531 |
| A0A1S4CN84 | 2.67986 | 0.00301 | 1.42215 |
| A0A1S4A1J2 | 2.69112 | 0.04275 | 1.42820 |
| A0A1S3XDY9 | 2.70427 | 0.00128 | 1.43524 |
| A0A1S3YTH6 | 2.70480 | 0.02101 | 1.43552 |
| A0A1S4CQL6 | 2.70532 | 0.00363 | 1.43580 |
| A0A1S4A1K3 | 2.71031 | 0.00146 | 1.43845 |
| A0A1S3ZVN0 | 2.73266 | 0.00200 | 1.45031 |
| A0A1S3ZBA7 | 2.74260 | 0.00735 | 1.45554 |
| P49332 | 2.75232 | 0.01449 | 1.46064 |
| A0A1S4A807 | 2.76228 | 0.00130 | 1.46586 |
| A0A1S4B7Z5 | 2.76333 | 0.00292 | 1.46641 |
| A0A1S4CXJ8 | 2.77787 | 0.02478 | 1.47397 |
| A0A1S3XC54 | 2.78382 | 0.00615 | 1.47706 |
| A0A1S3XUT0 | 2.79759 | 0.03602 | 1.48418 |
| A0A1S3YDT9 | 2.83075 | 0.00680 | 1.50118 |
| A0A1S4D550 | 2.83518 | 0.03776 | 1.50344 |
| A0A1S4CFE9 | 2.85063 | 0.03166 | 1.51128 |
| A0A059TCI1 | 2.91175 | 0.00046 | 1.54188 |
| A0A1S4CNW3 | 2.92566 | 0.01068 | 1.54876 |
| A0A1S3Y381 | 2.94292 | 0.00080 | 1.55725 |
| A0A1S4BYR7 | 2.94358 | 0.01209 | 1.55757 |
| A0A1S3XQ21 | 2.94525 | 0.01195 | 1.55839 |
| A0A1S4ATK9 | 2.96380 | 0.00283 | 1.56744 |
| A0A1S4CD84 | 2.98805 | 0.00740 | 1.57920 |
| A0A1S3YL77 | 3.00402 | 0.02724 | 1.58689 |
| Q0PWS5 | 3.00814 | 0.01221 | 1.58887 |
| A0A1S4D985 | 3.00877 | 0.01228 | 1.58917 |
| U3PVV5 | 3.01849 | 0.00932 | 1.59383 |
| P35476 | 3.02206 | 0.02593 | 1.59553 |
| A0A1S3ZX95 | 3.03271 | 0.01687 | 1.60061 |
| A0A1S3ZHH0 | 3.13872 | 0.00527 | 1.65018 |
| A0A1S3Z000 | 3.14048 | 0.00082 | 1.65098 |
| A0A1S3XVT6 | 3.21682 | 0.00243 | 1.68563 |
| A0A1S3X846 | 3.22231 | 0.00095 | 1.68809 |
| A0A1S3YU27 | 3.22244 | 0.00368 | 1.68815 |
| A0A1S4BFR8 | 3.25225 | 0.00644 | 1.70144 |
| A0A1S3XCV5 | 3.31578 | 0.00038 | 1.72934 |
| A0A1S3ZJW3 | 3.32729 | 0.01855 | 1.73435 |
| A0A1S4BG25 | 3.36734 | 0.03281 | 1.75161 |
| A0A1S4AR13 | 3.40166 | 0.00445 | 1.76624 |
| A0A1S3YZQ5 | 3.42653 | 0.02910 | 1.77674 |
| A0A1S3ZSC3 | 3.51138 | 0.00233 | 1.81204 |
| A0A1S3XUA2 | 3.51387 | 0.00109 | 1.81306 |
| A0A1S4B454 | 3.54039 | 0.00112 | 1.82391 |
| A0A1S4BWJ2 | 3.57127 | 0.00274 | 1.83644 |
| A0A1S4AFM9 | 3.67833 | 0.00001 | 1.87905 |
| A0A1S3YBX5 | 3.68443 | 0.03735 | 1.88144 |
| A0A1S4BZT5 | 3.69611 | 0.03004 | 1.88600 |
| B5AQ04 | 3.73768 | 0.01357 | 1.90214 |
| Q0PWS6 | 3.79086 | 0.00746 | 1.92252 |
| A0A1S3X831 | 3.87173 | 0.00018 | 1.95298 |
| A0A1S4C138 | 3.91427 | 0.04236 | 1.96874 |
| A0A1S4BPZ1 | 3.91752 | 0.00436 | 1.96994 |
| P35477 | 3.94813 | 0.02078 | 1.98117 |
| A0A1S3Z6I3 | 4.06040 | 0.01769 | 2.02162 |
| A0A1S4C053 | 4.10030 | 0.00121 | 2.03573 |
| A0A1S3Y6G2 | 4.18980 | 0.00766 | 2.06688 |
| A0A1S4BSH6 | 4.19835 | 0.00061 | 2.06982 |
| A0A1S4BCD1 | 4.24192 | 0.00364 | 2.08471 |
| A0A1S4CTH3 | 4.53728 | 0.02177 | 2.18182 |
| A0A1S3YZK4 | 4.71970 | 0.03185 | 2.23869 |
| A1KYB0 | 4.93901 | 0.01038 | 2.30422 |
| A0A1S3YSD8 | 5.07941 | 0.00357 | 2.34466 |
| A0A1S4D1U6 | 5.29513 | 0.00635 | 2.40466 |
| L0SS91 | 5.36098 | 0.00001 | 2.42249 |
| A0A1S3YS63 | 6.06462 | 0.02444 | 2.60041 |
| A0A1S3YYS8 | 6.23783 | 0.00920 | 2.64104 |

Table S2 DEPs localized in the chloroplast, cytoplasm and nucleus of cigar tobacco leaves in ST and NST groups

| Subcellular localization | Protein |
| --- | --- |
| chloroplast protein | A0A075M5G1, A0A0K1DA86, A0A140G1R1, A0A1S3WYR8, A0A1S3X094, A0A1S3X312, A0A1S3X353, A0A1S3X831, A0A1S3XCF6, A0A1S3XDY9, A0A1S3XFW0, A0A1S3XP23, A0A1S3XPR7, A0A1S3XQ21, A0A1S3XRS7, A0A1S3XUT6, A0A1S3XVI5, A0A1S3Y2C2, A0A1S3Y4F3, A0A1S3YA92, A0A1S3YDT9, A0A1S3YPR8, A0A1S3YUU3, A0A1S3YX82, A0A1S3YZ60, A0A1S3Z496, A0A1S3ZLD2, A0A1S3ZMD4, A0A1S3ZSC3, A0A1S3ZTX1, A0A1S3ZZ18, A0A1S4A6S1, A0A1S4A8Y8, A0A1S4AE99, A0A1S4AG60, A0A1S4AJJ4, A0A1S4AR72, A0A1S4AWM3, A0A1S4AZ56, A0A1S4BAT9, A0A1S4BID9, A0A1S4BMA4, A0A1S4BW60, A0A1S4BXK4, A0A1S4C9W7, A0A1S4CBR5, A0A1S4CF99, A0A1S4CN84, A0A1S4CST9, A0A1S4D3R4, A0A1S4D4V1, A0A1S4D6P6, A0A1S4D6R7, A0A1S4DFZ6, D3W9H9, K0I7G7, O24163, P09043, Q5M9Z2, Q5ZQU1, Q84QE5, Q8H6P9 |
| cytoplasm protein | A0A076L1Y6, A0A1S3WXL4, A0A1S3X4S6, A0A1S3XHG4, A0A1S3XIZ9, A0A1S3XKJ8, A0A1S3XLH4, A0A1S3XTK5, A0A1S3XW43, A0A1S3Y2E3, A0A1S3Y3M5, A0A1S3Y8G8, A0A1S3YBX5, A0A1S3YEW3, A0A1S3YIW7, A0A1S3YSD8, A0A1S3YY31, A0A1S3ZA83, A0A1S3ZFR9, A0A1S3ZR63, A0A1S3ZZC5, A0A1S4A0F9, A0A1S4A6K1, A0A1S4A731, A0A1S4AG17, A0A1S4AL80, A0A1S4ANZ2, A0A1S4AUA5, A0A1S4BAJ5, A0A1S4BBB3, A0A1S4BJ07, A0A1S4BQT4, A0A1S4BZQ6, A0A1S4C6E8, A0A1S4CGB1, A0A1S4CHI3, A0A1S4CHR0, A0A1S4CL28, A0A1S4CX79, A0A1S4CZ48, A0A1S4D364, A0A1S4D6L6, A0A1S4D980, A0A1S4DE74, A0A1S4DFK1, A0A1S4DGZ7, A0A1S4DRW2, D4P3S0, Q1KV12, Q6IVK8, Q9ZNW3 |
| nucleus protein | A0A1S3WXV3, A0A1S3WXX8, A0A1S3X130, A0A1S3X463, A0A1S3XAR5, A0A1S3XKN4, A0A1S3XRF1, A0A1S3XS20, A0A1S3XTZ2, A0A1S3XXL6, A0A1S3Y108, A0A1S3Y381, A0A1S3Y5Y3, A0A1S3YFN1, A0A1S3YHZ6, A0A1S3YJV1, A0A1S3YU27, A0A1S3YVD9, A0A1S3Z7F9, A0A1S3Z9K1, A0A1S3ZBF0, A0A1S3ZDY8, A0A1S3ZJS0, A0A1S3ZY65, A0A1S4A0W1, A0A1S4A845, A0A1S4A939, A0A1S4AEZ8, A0A1S4AFM9, A0A1S4APL7, A0A1S4BJ90, A0A1S4BPK4, A0A1S4BPN0, A0A1S4CDG7, A0A1S4CDH7, A0A1S4CIH1, A0A1S4D274, A0A1S4DKM6, A0A1S4DL73, A0A1S4DMW4, A0A1S4DNE4, A0A1S4DQ26, A0A9R6, D6PZY5, I2G7N7, Q8S939 |

Table S3 Information of metabolites analyzed in this study.

| Name | Kegg_ID | HMDB_ID | Lipidmaps_ID | FC | Pvalue | log2FC |
| --- | --- | --- | --- | --- | --- | --- |
| Raddeanin A | -- | HMDB0257094 | -- | 0.12418 | 0.00001 | -3.00944 |
| Ophiopogonin D | cpd:C17042 | -- | LMST01080113 | 0.15129 | 0.00012 | -2.72453 |
| TKK | -- | -- | -- | 0.16123 | 0.00001 | -2.63272 |
| N-p-Coumaroylspermidine | -- | -- | -- | 0.17032 | 0.00178 | -2.55365 |
| Arctiin | cpd:C16915 | HMDB0248568 | -- | 0.17052 | 0.00001 | -2.55199 |
| 4-methoxyphenyl 3,5-dimethyl-1-phenyl-1H-pyrazole-4-carboxylate | -- | -- | -- | 0.17417 | 0.00016 | -2.52137 |
| Geranylgeranyl pyrophosphate | cpd:C00353 | HMDB0004486 | -- | 0.28424 | 0.01239 | -1.8148 |
| Dictamnine | cpd:C10660 | HMDB0251209 | -- | 0.30194 | 0.00961 | -1.72763 |
| Cyclamic acid | -- | HMDB0031340 | -- | 0.33303 | 0.00191 | -1.58626 |
| Palmaturbine | -- | -- | -- | 0.35342 | 0.00529 | -1.50052 |
| 2,4-dimethyl-2,3-dihydrochromeno[4,3-c]pyrazol-3-one | -- | -- | -- | 0.36089 | 0.00225 | -1.47036 |
| N-Feruloyl putrescine | -- | -- | -- | 0.37773 | 0.02669 | -1.40457 |
| N-Isovaleroylglycine | -- | HMDB0000678 | -- | 0.38152 | 0.00251 | -1.39015 |
| Aldosterone | cpd:C01780 | HMDB0000037 | LMST02030026 | 0.38873 | 0.00673 | -1.36316 |
| YPH | -- | -- | -- | 0.39659 | 0.00919 | -1.33425 |
| Tracheloside | -- | HMDB0030557 | -- | 0.40274 | 0.00198 | -1.31206 |
| DGDG O-15:1_2:0 | -- | -- | -- | 0.40448 | 0.00653 | -1.30586 |
| 2-chloro-6-[(2-oxoazepan-3-yl)amino]benzonitrile | -- | -- | -- | 0.41565 | 0.04875 | -1.26655 |
| alpha-Hederin | cpd:C08954 | HMDB0248219 | -- | 0.42330 | 0.00047 | -1.24024 |
| Alpha-Mangostin | -- | HMDB0035796 | -- | 0.42625 | 0.00084 | -1.23023 |
| Proline | cpd:C16435 | HMDB0003411 | -- | 0.44300 | 0.03369 | -1.1746 |
| Dl-3-Hydroxynorvaline | -- | -- | -- | 0.44300 | 0.03369 | -1.1746 |
| Alantolactone | cpd:C09289 | HMDB0035906 | LMPR0103190013 | 0.45022 | 0.00076 | -1.15127 |
| Resiniferatoxin | cpd:C09179 | HMDB0242596 | -- | 0.45286 | 0.01795 | -1.14285 |
| N-Acetyl-DL-tryptophan | -- | -- | -- | 0.45514 | 0.00120 | -1.13561 |
| Sophocarpine | -- | HMDB0258379 | -- | 0.46873 | 0.01475 | -1.09315 |
| 1-Naphthyl acetate | -- | HMDB0243958 | -- | 0.47174 | 0.00770 | -1.08392 |
| Taurochenodeoxycholic Acid (sodium salt) | -- | -- | -- | 0.49334 | 0.01111 | -1.01933 |
| Lanatoside C | cpd:C13470 | -- | -- | 0.49823 | 0.00800 | -1.00509 |
| Tyrosylalanine | -- | HMDB0029098 | -- | 0.49910 | 0.02504 | -1.00259 |
| N-Acetyl-DL-glutamic acid | -- | -- | -- | 0.50257 | 0.00209 | -0.99258 |
| 11-keto Testosterone (CRM) | -- | -- | -- | 0.51583 | 0.01112 | -0.95502 |
| 5-Hydroxytryptophan | cpd:C01017 | HMDB0000472 | -- | 0.51783 | 0.00447 | -0.94943 |
| Kuwanon A | -- | HMDB0029505 | LMPK12110918 | 0.51957 | 0.02992 | -0.9446 |
| VQH | -- | -- | -- | 0.52200 | 0.01779 | -0.93787 |
| N1-(3-amino-4-chlorophenyl)-2-[2,4-di(tert-pentyl)phenoxy]acetamide | -- | -- | -- | 0.53434 | 0.00295 | -0.90416 |
| Ala-trp | -- | HMDB0013209 | -- | 0.53502 | 0.03804 | -0.90234 |
| QLH | -- | -- | -- | 0.53704 | 0.01795 | -0.8969 |
| Vardenafil N-oxide | -- | -- | -- | 0.53711 | 0.03707 | -0.89671 |
| D-Proline | cpd:C00763 | HMDB0003411 | -- | 0.53719 | 0.03088 | -0.8965 |
| LLK | -- | -- | -- | 0.54335 | 0.00426 | -0.88003 |
| tert-Butyl N-[1-(aminocarbonyl)-3-methylbutyl]carbamate | -- | -- | -- | 0.54374 | 0.00034 | -0.87899 |
| Maltotriitol | -- | -- | -- | 0.55522 | 0.03123 | -0.84886 |
| Isoguanosine | cpd:C08432 | -- | -- | 0.55579 | 0.02771 | -0.84737 |
| Guanosine | cpd:C00387 | HMDB0000133 | -- | 0.55998 | 0.01791 | -0.83655 |
| Yuheinoside | -- | -- | -- | 0.56027 | 0.03563 | -0.8358 |
| (+)-ar-Turmerone | -- | HMDB0035612 | -- | 0.56656 | 0.00111 | -0.81968 |
| L-Histidine | cpd:C00135 | HMDB0000177 | -- | 0.56798 | 0.01274 | -0.81607 |
| 8-iso-15-keto Prostaglandin F2α | -- | -- | -- | 0.56843 | 0.00047 | -0.81493 |
| 3-n-Butylphathlide | -- | -- | -- | 0.57910 | 0.00971 | -0.78812 |
| 2-Hydroxyhippuric acid | -- | HMDB0000840 | -- | 0.58305 | 0.04005 | -0.77829 |
| 3-[4-methyl-1-(2-methylpropanoyl)-3-oxocyclohexyl]butanoic acid | -- | -- | -- | 0.58932 | 0.00729 | -0.76287 |
| Myristoleic Acid | cpd:C08322 | HMDB0002000 | LMFA01030051 | 0.60613 | 0.03555 | -0.7223 |
| (5E)-7-methylidene-10-oxo-4-(propan-2-yl)undec-5-enoic acid | -- | -- | -- | 0.60881 | 0.01011 | -0.71592 |
| Ala-Ile | -- | HMDB0028690 | -- | 0.60989 | 0.00001 | -0.71337 |
| FAHFA 2:0/18:1 | -- | -- | -- | 0.62884 | 0.00513 | -0.66924 |
| Guanine | cpd:C00242 | HMDB0000132 | -- | 0.64137 | 0.02812 | -0.64075 |
| Aucubin | cpd:C09771 | HMDB0036562 | LMPR0102070006 | 0.64476 | 0.00941 | -0.63316 |
| TLK | -- | -- | -- | 0.64532 | 0.01563 | -0.6319 |
| Sedanolide | -- | HMDB0302242 | -- | 0.64625 | 0.00986 | -0.62983 |
| 17(S)-HpDHA | -- | -- | -- | 0.64812 | 0.00701 | -0.62565 |
| 1-(3,4-dihydroxyphenyl)-7-(4-hydroxyphenyl)heptan-3-one | -- | -- | -- | 0.64934 | 0.04305 | -0.62294 |
| ALK | -- | -- | -- | 0.65045 | 0.00172 | -0.62047 |
| 1,1-Dimethyl-2-oxopropyl N-[2-(2-pyridyl)ethyl]carbamate | -- | -- | -- | 0.65528 | 0.02621 | -0.60982 |
| LPG O-16:3 | -- | -- | -- | 0.65773 | 0.00655 | -0.60442 |
| 3-Furoic acid | -- | HMDB0000444 | -- | 0.66876 | 0.00036 | -0.58043 |
| SPK | -- | -- | -- | 0.67129 | 0.00080 | -0.57498 |
| Luteolin | cpd:C01514 | HMDB0005800 | LMPK12110006 | 0.67355 | 0.00305 | -0.57013 |
| Ajugol | -- | HMDB0248074 | -- | 0.67473 | 0.00739 | -0.56761 |
| gamma-Glutamylleucine | -- | HMDB0011171 | -- | 0.67813 | 0.03949 | -0.56035 |
| 4-Oxoproline | cpd:C01877 | HMDB0304793 | -- | 0.67933 | 0.04164 | -0.5578 |
| N-Methyltryptamine | cpd:C06213 | HMDB0004370 | -- | 0.68263 | 0.00206 | -0.55082 |
| (S)-(+)-2-(anilinomethyl) pyrrolidine | -- | -- | -- | 0.69509 | 0.00033 | -0.52473 |
| 13-HPODE | -- | HMDB0003871 | LMFA02000034 | 0.69854 | 0.00582 | -0.51758 |
| N-p-Coumaroyl putrescine | -- | -- | -- | 0.70308 | 0.02775 | -0.50823 |
| (2R)-2,3-Dihydroxypropanoic acid | -- | HMDB0000148 | -- | 0.70615 | 0.01186 | -0.50194 |
| Dinophysistoxin-1 | -- | HMDB0030442 | -- | 0.70699 | 0.02087 | -0.50022 |
| Eurycomalactone | cpd:C08759 | -- | -- | 0.71156 | 0.04597 | -0.49093 |
| Fraxinol | -- | -- | -- | 0.71551 | 0.00846 | -0.48294 |
| Hordatine B | cpd:C08308 | HMDB0030459 | -- | 0.71665 | 0.02798 | -0.48065 |
| Hirsuteine | cpd:C16971 | -- | -- | 0.72382 | 0.01615 | -0.46629 |
| N-Desmethylclobazam | -- | HMDB0060970 | -- | 0.72779 | 0.01846 | -0.4584 |
| N-Acetyl-L-phenylalanine | cpd:C03519 | HMDB0000512 | -- | 0.73308 | 0.01881 | -0.44796 |
| VLH | -- | -- | -- | 0.73765 | 0.01422 | -0.43898 |
| Kaempferol | cpd:C05903 | HMDB0005801 | LMPK12110003 | 0.74391 | 0.04275 | -0.42680 |
| D-(-)-Lyxose | -- | -- | -- | 0.74684 | 0.01577 | -0.42112 |
| 2-(tert-butyl)-6,7-dimethoxy-4H-3,1-benzoxazin-4-one | -- | -- | -- | 0.74731 | 0.00364 | -0.42021 |
| Gramine | cpd:C08304 | -- | -- | 0.74792 | 0.00624 | -0.41904 |
| 1-[(3,5-dimethylisoxazol-4-yl)sulfonyl]piperidine | -- | -- | -- | 0.75431 | 0.01401 | -0.40677 |
| 2-Furoic acid | cpd:C01546 | HMDB0000617 | -- | 0.75657 | 0.00934 | -0.40244 |
| 3-[3-(beta-D-Glucopyranosyloxy)-2-methoxyphenyl]propanoic acid | -- | -- | -- | 0.75661 | 0.01626 | -0.40237 |
| RMK | -- | -- | -- | 0.76699 | 0.01802 | -0.3827 |
| Alanyltyrosine | -- | HMDB0028699 | -- | 0.76963 | 0.04865 | -0.37776 |
| Pipecolic acid | cpd:C00408 | HMDB0000070 | -- | 0.78614 | 0.02801 | -0.34714 |
| S-Adenosylhomocysteine | cpd:C00021 | HMDB0000939 | -- | 0.78631 | 0.02227 | -0.34683 |
| 3-Indoleacetonitrile | cpd:C02938 | HMDB0006524 | -- | 0.78697 | 0.00388 | -0.34562 |
| 2-Hydroxyphenylalanine | -- | HMDB0006050 | -- | 0.78910 | 0.04485 | -0.34171 |
| 3-(3,4-dihydroxyphenyl)propanoic acid | -- | HMDB0000423 | -- | 0.79367 | 0.02071 | -0.33338 |
| Chlorogenic acid methyl ester | -- | -- | -- | 0.79488 | 0.04635 | -0.33117 |
| N-Acetyl-D-alloisoleucine | -- | -- | -- | 0.79639 | 0.04415 | -0.32845 |
| Sinapaldehyde glucoside | -- | -- | -- | 0.79848 | 0.01511 | -0.32467 |
| 6-Methylquinoline | -- | HMDB0033115 | -- | 0.81554 | 0.03115 | -0.29417 |
| Benzamidine | cpd:C01784 | HMDB0248970 | -- | 0.82185 | 0.00176 | -0.28304 |
| 5-Methoxysalicylic acid | -- | HMDB0001868 | -- | 0.82680 | 0.00926 | -0.27439 |
| 5,6-Dimethylbenzimidazole | cpd:C03114 | HMDB0003701 | -- | 0.82789 | 0.00239 | -0.27248 |
| ethyl 4-[(6-methyl-3-pyridazinyl)oxy]benzoate | -- | -- | -- | 0.83093 | 0.04678 | -0.26719 |
| 3'-Hydroxypterostilbene | -- | -- | -- | 1.20755 | 0.02138 | 0.27208 |
| 1-(4-butylphenyl)-3-(dimethylamino)propan-1-one hydrochloride | -- | -- | -- | 1.20950 | 0.01447 | 0.27441 |
| SQH | -- | -- | -- | 1.22781 | 0.01985 | 0.29609 |
| Praeruptorin A | -- | HMDB0242259 | -- | 1.23779 | 0.04235 | 0.30777 |
| s7p | -- | -- | -- | 1.23805 | 0.02440 | 0.30808 |
| Elaidic acid | cpd:C01712 | HMDB0000573 | -- | 1.24406 | 0.03365 | 0.31505 |
| Hippuric Acid | cpd:C01586 | HMDB0000714 | -- | 1.24862 | 0.02883 | 0.32034 |
| LPI 18:3 | -- | -- | LMGP06050028 | 1.24974 | 0.00634 | 0.32163 |
| 2,4-Dimethylbenzaldehyde | -- | HMDB0032142 | -- | 1.25491 | 0.00725 | 0.32758 |
| N-lactoyl-phenylalanine | -- | HMDB0062175 | -- | 1.26584 | 0.01800 | 0.34009 |
| Mevalonic acid | cpd:C00418 | HMDB0000227 | LMFA01050352 | 1.26605 | 0.03288 | 0.34034 |
| 2,3-dihydroxypropyl 12-methyltridecanoate | -- | -- | -- | 1.26625 | 0.02139 | 0.34057 |
| Indole-3-pyruvic acid | -- | HMDB0060484 | -- | 1.28195 | 0.01393 | 0.35834 |
| Hydroxysafflor yellow A | -- | HMDB0040677 | -- | 1.28229 | 0.03060 | 0.35873 |
| (3R)-4,4-Dimethyl-2-oxotetrahydro-3-furanyl beta-D-glucopyranoside | -- | -- | -- | 1.28306 | 0.04698 | 0.35958 |
| SNH | -- | -- | -- | 1.28427 | 0.03594 | 0.36095 |
| Sclareolide | -- | HMDB0035293 | -- | 1.28649 | 0.02533 | 0.36344 |
| 3-[(5-nitropyridin-2-yl)oxy]-1H-indazole | -- | -- | -- | 1.29296 | 0.01301 | 0.37068 |
| TNH | -- | -- | -- | 1.29370 | 0.02678 | 0.37150 |
| (2R,3S,4S,5R,6R)-2-(hydroxymethyl)-6-(2-phenylethoxy)oxane-3,4,5-triol | -- | -- | -- | 1.29691 | 0.01564 | 0.37508 |
| Docosahexaenoic acid | cpd:C06429 | HMDB0002183 | LMFA01031176 | 1.30038 | 0.00468 | 0.37893 |
| Ambroxane | -- | -- | -- | 1.32628 | 0.00365 | 0.40738 |
| Geniposidic acid | cpd:C11673 | HMDB0034942 | LMPR0102070043 | 1.33700 | 0.02403 | 0.41900 |
| 3-Indolepropionic acid | -- | HMDB0002302 | -- | 1.33734 | 0.01084 | 0.41937 |
| Citral | -- | HMDB0035092 | -- | 1.33905 | 0.01491 | 0.42121 |
| Ixoside | -- | -- | LMPR0102070040 | 1.34533 | 0.00643 | 0.42796 |
| Choline | cpd:C00114 | HMDB0000097 | -- | 1.37042 | 0.02672 | 0.45462 |
| (±)18-HEPE | -- | -- | -- | 1.37387 | 0.02690 | 0.45825 |
| Palmitic acid | cpd:C00249 | HMDB0000220 | LMFA01010001 | 1.38242 | 0.03518 | 0.46720 |
| D-(+)-Camphor | -- | HMDB0059838 | -- | 1.38600 | 0.03060 | 0.47093 |
| LPS 18:3 | -- | -- | LMGP03050029 | 1.38661 | 0.00125 | 0.47156 |
| Diacetoxyscirpenol | cpd:C09662 | HMDB0035104 | -- | 1.38688 | 0.02004 | 0.47184 |
| 2-piperidinobenzoic acid | -- | -- | -- | 1.38843 | 0.01500 | 0.47345 |
| 2,4-dihydroxyheptadec-16-en-1-yl acetate | -- | -- | LMFA05000640 | 1.39056 | 0.04871 | 0.47566 |
| DL-Stachydrine | -- | -- | -- | 1.39595 | 0.01588 | 0.48125 |
| Carvone | cpd:C11383 | HMDB0035824 | -- | 1.40247 | 0.03247 | 0.48797 |
| 6-Methylcoumarin | -- | HMDB0032394 | -- | 1.40361 | 0.02367 | 0.48914 |
| Heroin-d3 | -- | -- | -- | 1.40975 | 0.00510 | 0.49544 |
| (-)-Norepinephrine N-hexoside | -- | -- | -- | 1.41512 | 0.03117 | 0.50092 |
| Polypodine B | cpd:C08834 | HMDB0302993 | LMST01010198 | 1.42087 | 0.00536 | 0.50677 |
| N6-Succinyl Adenosine | -- | HMDB0255303 | -- | 1.42266 | 0.00288 | 0.50859 |
| N,N'-di[4-(2,6-dimethylmorpholino)phenyl]thiourea | -- | -- | -- | 1.42379 | 0.02876 | 0.50974 |
| Salidroside | cpd:C06046 | HMDB0257463 | -- | 1.42399 | 0.03864 | 0.50994 |
| LPS 18:2 | -- | -- | LMGP03050011 | 1.42417 | 0.00114 | 0.51012 |
| Columbianadin | -- | HMDB0250407 | -- | 1.42417 | 0.02768 | 0.51013 |
| beta-Caryophyllene | cpd:C09629 | HMDB0036792 | LMPR0103120001 | 1.42869 | 0.03658 | 0.51469 |
| octadec-9-ynoic acid | -- | -- | -- | 1.43122 | 0.01262 | 0.51724 |
| 3'-Adenosine monophosphate (3'-AMP) | -- | -- | -- | 1.43395 | 0.04264 | 0.51999 |
| Phenylacetylglutamine | cpd:C04148 | HMDB0006344 | -- | 1.43754 | 0.02624 | 0.52360 |
| 2-Isopropylmalic acid | cpd:C02504 | HMDB0000402 | -- | 1.43941 | 0.01278 | 0.52548 |
| Azetidine-2-carboxylic acid | -- | HMDB0248797 | -- | 1.44015 | 0.00161 | 0.52621 |
| N'-[4-(trifluoromethyl)benzoyl]-6-quinoxalinecarbohydrazide | -- | -- | -- | 1.44732 | 0.02477 | 0.53338 |
| Madecassic acid | -- | HMDB0036670 | -- | 1.45097 | 0.01004 | 0.53702 |
| Tyramine | cpd:C00483 | HMDB0000306 | -- | 1.46359 | 0.00953 | 0.54951 |
| Geraniol | cpd:C01500 | HMDB0005812 | -- | 1.46432 | 0.01233 | 0.55023 |
| Docosatrienoic acid | -- | HMDB0002823 | -- | 1.46518 | 0.02335 | 0.55107 |
| 2-hydroxy-6-[(8Z,11Z)-pentadeca-8,11,14-trien-1-yl]benzoic acid | -- | -- | -- | 1.46813 | 0.03651 | 0.55398 |
| Sesamoside | -- | -- | -- | 1.46827 | 0.02677 | 0.55412 |
| N~5~-(1,3,5-trimethyl-1H-pyrazol-4-yl)-1H-1,2,4-triazole-3,5-diamine | -- | -- | -- | 1.47639 | 0.01015 | 0.56207 |
| Acetophenone | cpd:C07113 | HMDB0033910 | -- | 1.47688 | 0.00265 | 0.56255 |
| D-Erythrose 4-phosphate | cpd:C00279 | HMDB0001321 | -- | 1.48339 | 0.00163 | 0.56890 |
| LPE 18:1 | -- | -- | LMGP02050064 | 1.48387 | 0.01922 | 0.56936 |
| Androsin | -- | -- | -- | 1.48467 | 0.04876 | 0.57014 |
| Epimedin C | -- | -- | LMPK12112015 | 1.49042 | 0.02253 | 0.57572 |
| 5-[(8Z,11Z)-pentadeca-8,11-dien-1-yl]benzene-1,3-diol | -- | -- | -- | 1.49181 | 0.03267 | 0.57706 |
| Ascorbic acid | cpd:C00072 | HMDB0000044 | -- | 1.49989 | 0.02304 | 0.58485 |
| LPC 18:2 | -- | -- | LMGP01050035 | 1.50572 | 0.02075 | 0.59045 |
| Steviolbioside | -- | HMDB0036707 | LMPR01040122 | 1.51811 | 0.02010 | 0.60228 |
| 4-(3-methoxy-5,6-dihydrobenzo[c]acridin-7-yl)morpholine | -- | -- | -- | 1.53318 | 0.00610 | 0.61653 |
| Wulignan A1 | -- | -- | -- | 1.53385 | 0.00439 | 0.61716 |
| LPG 18:3 | -- | -- | LMGP04050032 | 1.53560 | 0.02592 | 0.61881 |
| 1,2-dihydroxyheptadec-16-yn-4-yl acetate | -- | -- | LMFA05000645 | 1.53888 | 0.00776 | 0.62188 |
| L-Dopa | cpd:C00355 | HMDB0000181 | -- | 1.54580 | 0.00026 | 0.62835 |
| (3beta,9xi)-3-(beta-D-Glucopyranosyloxy)-14-hydroxycard-20(22)-enolide | -- | -- | -- | 1.54887 | 0.01119 | 0.63122 |
| LPC 18:1-SN1 | -- | -- | -- | 1.56087 | 0.03687 | 0.64235 |
| LPG 16:0 | -- | -- | LMGP04050008 | 1.58171 | 0.02529 | 0.66148 |
| Euphorbia factor L1 | -- | -- | -- | 1.58701 | 0.03548 | 0.66631 |
| N', N''-DiFeruloylspermidine | -- | -- | -- | 1.58751 | 0.00161 | 0.66677 |
| Epimedin B | -- | HMDB0251854 | LMPK12112011 | 1.58889 | 0.00444 | 0.66802 |
| Stearamide | cpd:C13846 | HMDB0034146 | LMFA08010003 | 1.60924 | 0.04129 | 0.68638 |
| Valepotriate | -- | HMDB0034493 | -- | 1.61102 | 0.03712 | 0.68798 |
| 4-(2,3-dihydro-1H-indol-1-yl)-1-phenyl-1H-pyrazolo[3,4-d]pyrimidine | -- | -- | -- | 1.61437 | 0.00114 | 0.69097 |
| 2-{[2-(4-methylpiperazino)phenyl]methylene}hydrazine-1-carbothioamide | -- | -- | -- | 1.61721 | 0.00268 | 0.69350 |
| Sinapyl Alcohol | cpd:C02325 | HMDB0013070 | -- | 1.62245 | 0.00407 | 0.69817 |
| PC O-18:2 | -- | -- | -- | 1.62586 | 0.00675 | 0.70120 |
| (R)-3-Hydroxy myristic acid | -- | -- | -- | 1.63029 | 0.01858 | 0.70512 |
| LPE 18:3 | -- | -- | LMGP02050043 | 1.63421 | 0.00113 | 0.70860 |
| Piceatannol | cpd:C05901 | HMDB0303377 | LMPK13090006 | 1.63959 | 0.01481 | 0.71333 |
| LPE 18:2 | -- | -- | LMGP02050041 | 1.64377 | 0.00756 | 0.71701 |
| Isorhapontigenin | -- | HMDB0303171 | -- | 1.65273 | 0.00707 | 0.72485 |
| 2-[5-(2-hydroxypropyl)oxolan-2-yl]propanoic acid | -- | -- | -- | 1.65336 | 0.00001 | 0.72540 |
| Trigoneoside Xb | -- | HMDB0036484 | -- | 1.66738 | 0.01491 | 0.73759 |
| Coenzyme Q2 | -- | HMDB0006709 | -- | 1.67735 | 0.01061 | 0.74618 |
| 1,N6-Etheno-2-Deoxy-Adenosine | -- | -- | -- | 1.68389 | 0.03255 | 0.75180 |
| Euphorbia factor L3 | -- | -- | -- | 1.70816 | 0.01183 | 0.77244 |
| N-[(Tert-butoxy)carbonyl]-L-tryptophan | -- | -- | -- | 1.70965 | 0.00086 | 0.77370 |
| 1-O-(3,4,5-Trimethoxybenzoyl)-beta-L-galactopyranose | -- | -- | -- | 1.71929 | 0.01060 | 0.78181 |
| 6-(3-hydroxybutan-2-yl)-5-(hydroxymethyl)-4-methoxy-2H-pyran-2-one | -- | -- | -- | 1.72457 | 0.00247 | 0.78624 |
| 6-Hydroxymelatonin | cpd:C05643 | HMDB0004081 | -- | 1.72944 | 0.01189 | 0.79031 |
| 1-Monopalmitin | -- | HMDB0245964 | -- | 1.75467 | 0.00251 | 0.81120 |
| Nodakenetin | cpd:C09278 | HMDB0302268 | -- | 1.75706 | 0.01731 | 0.81316 |
| Praeruptorin E | -- | -- | -- | 1.77047 | 0.00751 | 0.82413 |
| LPC 18:3 | -- | -- | LMGP01050128 | 1.77805 | 0.00179 | 0.83030 |
| LPC 18:3-SN1 | -- | -- | -- | 1.78482 | 0.00075 | 0.83578 |
| Epinastine | -- | HMDB0014889 | -- | 1.79120 | 0.00703 | 0.84092 |
| 5,6-dimethyl-3-[5-(trifluoromethyl)pyridin-2-yl]-1,2,4-triazine | -- | -- | -- | 1.80176 | 0.01416 | 0.84941 |
| PC O-18:1 | -- | -- | LMGP01020147 | 1.81073 | 0.01611 | 0.85657 |
| 4-acetyl-4-(ethoxycarbonyl)heptanedioic acid | -- | -- | -- | 1.84411 | 0.00149 | 0.88292 |
| N-Sinapoylputrescine | -- | -- | -- | 1.88685 | 0.00095 | 0.91598 |
| Momordin Ic | -- | -- | -- | 1.90732 | 0.00319 | 0.93155 |
| LPG 15:1 | -- | -- | LMGP04050018 | 1.90757 | 0.02481 | 0.93173 |
| Narasin | -- | HMDB0030448 | -- | 1.91745 | 0.03781 | 0.93919 |
| Phosphocholine | cpd:C00588 | HMDB0001565 | -- | 1.96841 | 0.00001 | 0.97703 |
| Heptamethoxyflavone | -- | -- | -- | 1.98081 | 0.02677 | 0.98609 |
| Schisantherin A | cpd:C10881 | HMDB0258170 | -- | 1.98755 | 0.01633 | 0.99099 |
| Morphine | cpd:C01516 | HMDB0014440 | -- | 1.98881 | 0.04591 | 0.99191 |
| LPA 17:1 | -- | -- | LMGP10050002 | 1.98912 | 0.00999 | 0.99213 |
| Norisoboldine | -- | HMDB0033357 | -- | 2.00153 | 0.03468 | 1.00110 |
| PC O-18:3 | -- | -- | -- | 2.06194 | 0.00051 | 1.04400 |
| 13,14-dihydro-15-keto-tetranor Prostaglandin E2 | -- | -- | -- | 2.08192 | 0.00540 | 1.05792 |
| 7-Methoxyflavone | -- | -- | LMPK12110021 | 2.11078 | 0.01061 | 1.07777 |
| LPG 18:4 | -- | -- | LMGP04050021 | 2.12962 | 0.00249 | 1.09060 |
| 4-Methylumbelliferone hydrate | -- | -- | -- | 2.16535 | 0.00099 | 1.11460 |
| Licoagroside D | -- | -- | -- | 2.20264 | 0.02731 | 1.13923 |
| TriacetonaMine | -- | HMDB0031179 | -- | 2.21252 | 0.01075 | 1.14569 |
| 8(9)-EET Ethanolamide | -- | -- | -- | 2.33479 | 0.00628 | 1.22329 |
| Phenmetrazine | cpd:C07432 | HMDB0014968 | -- | 2.39546 | 0.00293 | 1.26030 |
| (2S)-Isoxanthohumol | -- | -- | -- | 2.53215 | 0.00651 | 1.34036 |
| Tetrahydropiperine | -- | -- | -- | 2.56259 | 0.00021 | 1.35760 |
| N alpha-Acetyl-L-Arginine | -- | -- | -- | 2.58785 | 0.00565 | 1.37175 |
| Tuberostemonine | -- | -- | -- | 2.60488 | 0.03839 | 1.38122 |
| Carbendazim | cpd:C10897 | HMDB0031769 | -- | 2.80407 | 0.01424 | 1.48752 |
| PDMP | -- | -- | -- | 2.85853 | 0.03979 | 1.51527 |
| Lancerin | cpd:C10075 | -- | -- | 2.99987 | 0.02033 | 1.58490 |
| N-({5-[(4-methylpiperazino)sulfonyl]-2-thienyl}methyl)benzamide | -- | -- | -- | 3.20450 | 0.00154 | 1.68010 |
| Puerarin | cpd:C10524 | HMDB0240265 | LMPK12050005 | 3.91112 | 0.01530 | 1.96758 |
| 5-methoxy-8,8-dimethyl-2-phenyl-4H,8H-pyrano[2,3-h]chromen-4-one | -- | -- | -- | 4.19366 | 0.00169 | 2.06821 |
| N-(Phenylacetyl)-L-phenylalanine | -- | HMDB0002372 | -- | 4.39383 | 0.00074 | 2.13548 |
| Milbemycin A3 oxime | -- | -- | -- | 4.69566 | 0.04063 | 2.23133 |
| methyl 2-morpholino-4-(trifluoromethyl)pyrimidine-5-carboxylate | -- | -- | -- | 4.82508 | 0.00017 | 2.27055 |
| Pilocarpine | cpd:C07474 | HMDB0015217 | -- | 4.95965 | 0.03585 | 2.31023 |
| Cyclobutyl fentanyl-d5 | -- | -- | -- | 6.03327 | 0.02033 | 2.59294 |
| 6-Chloronicotinic acid | -- | HMDB0247049 | -- | 8.74545 | 0.00001 | 3.12853 |
| 2-Chloro-4,6-dimethylaniline | -- | -- | -- | 12.98000 | 0.00197 | 3.69821 |
| 2-chloro-N'-(4-chlorophenyl)-6-methylisonicotinohydrazide | -- | -- | -- | 14.83041 | 0.00023 | 3.89048 |
| Nodularin | cpd:C15713 | HMDB0255692 | -- | 17.14730 | 0.00043 | 4.09991 |
| Milbemycin A4 oxime | -- | -- | -- | 23.04388 | 0.00081 | 4.52631 |
| 4-Hydroxybenzophenone | cpd:C14230 | HMDB0240708 | -- | 41.70433 | 0.00001 | 5.38212 |
